# Kinetically Controlled Morphologies of Magnetic Nanoparticles through Ligand and Precursor Chemistry

**DOI:** 10.1021/acsnanoscienceau.5c00099

**Published:** 2025-11-05

**Authors:** Rabia Amin, Yihao Wang, Johannes Berlin, Markus Etzkorn, Christopher R. Everett, Susanne Kempter, Meinhard Schilling, Peter Müller-Buschbaum, Jan Lipfert, Mohammad Suman Chowdhury, Aidin Lak

**Affiliations:** † Institute for Electrical Measurement Science and Fundamental Electrical Engineering and Laboratory for Emerging Nanometrology (LENA), Hans-Sommer-Str. 66, Braunschweig 38106, Germany; ‡ Institute of Physics, University of Augsburg, Universitätsstraße 1, Augsburg 86159, Germany; § Institute of Applied Physics, 26527 TU Braunschweig, Mendelssohnstraße 2, Braunschweig 38106, Germany; ∥ TUM School of Natural Sciences, Department of Physics, Chair for Functional Materials, 9184 Technical University of Munich, James-Franck Str. 1, Garching 85748, Germany; ⊥ Department of Physics and Center for NanoScience, LMU Munich, Amalienstrasse 54, Munich 80539, Germany

**Keywords:** cobalt zinc ferrite magnetic nanoparticles, capping ligands, precursor chemistry, facet-selective growth, tetrahedral nanoparticles, rod-shaped nanoparticles

## Abstract

Kinetically controlled morphologies of colloidal magnetic nanoparticles possess unique magnetic properties, making them highly promising for applications in magnetogenetics as magnetic torque probes. Yet, their size-controlled chemical synthesis is in its nascent state. Here, we present a capping-ligand-directed approach to tune the morphology and magnetic properties of Co_*x*_Zn_*y*_Fe_3‑(*x*+*y*)_O_4_ nanoparticles by adding sodium oleate as a cocapping ligand to oleic acid during synthesis, resulting in the formation of monodisperse tetrahedral nanoparticles. Increasing the molar ratio of sodium oleate to oleic acid promotes facet-selective passivation along {111} facets, leading to progressive truncation of tetrahedra and yielding morphologies ranging from truncated tetrahedra to extremely truncated rod-like shapes. Our electron microscopy studies show that the synthesis of tetrahedron-shaped nanoparticles does not require a symmetry-breaking transformation from octahedra, as the initial crystallite formed is tetrahedra. When sodium oleate is removed from the synthesis, thermodynamically driven monodisperse octahedral nanoparticles are formed. We find that ligand composition also influences the doping of ions into the crystal structure, with higher sodium oleate concentrations reducing Zn^2+^ incorporation due to modified metal–ligand coordination. Tetrahedral nanoparticles synthesized under optimal conditions exhibit the highest room temperature saturation magnetization among other morphologies, highlighting their potential for magnetic-nanoparticle-based biosensing applications. Our study underscores that not only morphology but also magnetic characteristics of nanoparticles can be tuned by a ligand-guided chemistry.

## Graphical abstract

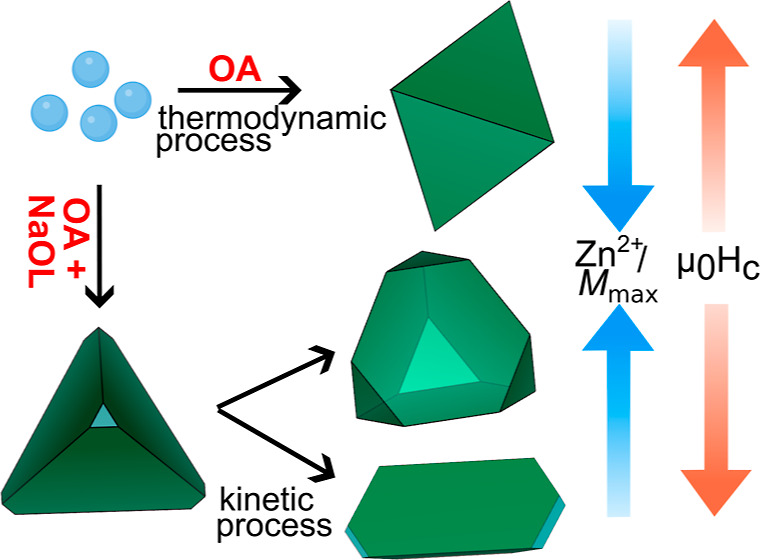

## Introduction

Magnetic nanomaterials have led to key technological advances in medicine, single-molecule spectroscopy, and bioimaging. Homogeneous magnetic bioassays for sensitive and extraction-free detection of nucleic acids and proteins^1–3^ and magneto-mechanical actuation of cellular receptors for noninvasive manipulation of cellular pathways^4,5^ are among the most appealing applications. These technologies require nanoparticles with finely tuned morphological, magnetic, colloidal, and surface properties. Controlling the morphology of colloidal magnetic nanoparticles (MNPs) is still a significant challenge,^6–9^ mainly due to the complexity of solution-phase synthesis, primarily due to the intricate roles of surface capping ligands. The nature and concentration of capping ligands govern the formation of metal-carboxylate precursors,^10^ nucleation, and growth of nanocrystals, all together dictating the final morphology of MNPs.

Prior studies have elucidated the formation of the iron-oleate (FeOL) precursor in the synthesis of iron oxide nanoparticles, revealing its tri-iron-oxo-carboxylate cluster structure.^10–13^ Predicting the precursor formation in mixed metal tricomponent MNPs is particularly challenging because the metal ions involved exhibit nearly identical precursor decomposition profiles, making it difficult to distinguish and control their individual contributions during synthesis.^14,15^ Reactions involving more than one metal precursor (M and Ḿ) often yield a mixture of different metal oxide nanoparticles (MO_*x*_ and ḾO_*y*_) rather than forming ternary oxide nanoparticles (NPs).^16,17^ This presents a significant challenge in understanding the ligand-driven precursor formation of mixed metal tricomponent MNPs, requiring a fundamental understanding of the precursor reaction chemistry and its influence on the final morphologies of MNPs. The nature and structure of the formed precursor not only determine its decomposition kinetics and monomer generation but also influence how capping ligands interact with specific crystal facets at later growth stages, thereby regulating growth kinetics and particle morphology.^18,19^

The synthesis of metal oxide NPs includes the coordination of carboxylate ligands with metal ions to form molecular precursors, followed by their decomposition to monomers, nucleation, and growth. The carboxylate metal-binding moieties include oxygen atoms that typically serve as oxygen sources for forming oxide NPs. According to the hard–soft acid base theory,^20^ carboxylates are hard bases, allowing them to strongly coordinate with hard metal ions such as Fe^3+^ on the surface of NPs. At elevated temperatures, the metal-coordinated carboxylate groups decompose through homolytic cleavage of metal–oxygen and carbon–oxygen bonds, generating short-lived radical species.^21^The chemical interactions among these transient intermediates promote the release of metal ions and facilitate a sustained monomer supply to the growing nuclei, thereby promoting NP growth.^21–23^

Beyond their role in precursor formation, capping ligands play a significant role in directing nanoparticle growth and regulating particle size by modifying surface free energies across different crystal facets and by interacting with metal atoms on the particle surface.^24^ In thermodynamically controlled processes, NP morphology is governed by the minimization of total surface energy, with facets of lower surface energy being more stable and thus more likely to appear in the final morphology.^25^ Capping ligands, when adsorbed on certain crystal facets, can pose a physical barrier to monomer deposition and diffusion, thus controlling the particle morphology via kinetically controlled processes.^25,26^ When capping ligands bind onto a crystal facet, they stabilize the surface atoms, thus passivating that crystal facet. In the subsequent growth stage, the growth rate of each facet determines its surface exposure in the final morphology: facets that are shielded by ligands experience slower growth and tend to persist, while those being weakly shielded grow more rapidly and eventually diminish.^27,28^ Thermodynamically driven morphologies such as spherical or cuboctahedron^,^ ^29,30^ NPs are the result of oleic acid (OA) with a similar binding affinity to all three primary low-index crystal facets.^27,29,31–33^ While several researchers have observed the formation of cubic MNPs when sodium oleate (NaOL) was added as a secondary ligand to the synthesis,^7,32,33^ few have reported MNPs with tetrahedron shapes by tuning the molar ratios of NaOL to FeOL.^28^ Looking into the literature, the role of NaOL as a capping ligand in directing the shape of NPs is still controversial. A slight change in the ratio of NaOL to FeOL has led to morphologies such as bipyramids,^32^ tetrahedrons,^28^ and discs.^34,35^ The formation of these out-of-equilibrium morphologies is unexpected, since iron oxide has a cubic crystal structure, which favors isotropic growth. Compared to its more symmetric counterpart, the octahedron, the surface of a tetrahedron is also covered by four {111} facets. However, the surface area to volume ratio of a tetrahedron is 1.3 times larger than that of an octahedron, which makes the tetrahedral shape less favorable in thermodynamically controlled synthesis.^36–38^ Tetrahedron-shaped NPs have been successfully synthesized for noble metals such as Pt,^37^ Au,^38,39^ Pd,^40^ Rh,^41^ and Ag,^42,43^ yet how metal oxides MNPs are grown into kinetically controlled morphologies is far less understood. This is primarily due to the reduced (*T*_d_) symmetry compared to the parent FCC lattice (O_h_).^36,37^ Although both octahedra and tetrahedra are fully exposed by {111} facets, the formation of tetrahedra requires the selective growth of only four symmetrically placed {111} facets out of eight equivalent possibilities. In an isotropic growth environment, with atoms depositing from all directions, there is no inherent mechanism to favor this reduced subset of growth orientations, making the formation of tetrahedra less likely.^37^ For this reason, the synthesis of tetrahedron-shaped MNPs, which are often observed as side products in several syntheses, has been elusive and rarely explored,^36,37^ despite two decades of research on colloidal MNPs.

Here, we explore the role of ligand-guided precursor formation in the nucleation and growth mechanism of mixed metal ferrite MNPs. We address the challenge of achieving selective facet stabilization during nucleation and growth, highlighting the crucial interplay among precursor chemistry, capping ligand interactions, and growth kinetics in determining MNP morphology. We show how subtle changes in precursor compositions, induced by ligand chemistry, critically govern the successful incorporation of metal ions in Co_*x*_Zn_*y*_Fe_3‑(*x*+*y*)_O_4_ MNPs. By adjusting the molar ratios of two regularly used ligands, OA and NaOL, we show a robust strategy to steer nanoparticle growth from well-defined thermodynamically driven morphologies to kinetically driven morphologies. Our study underscores the crucial role of ligand chemistry in directing MNP growth with properties tailored for bioapplications, particularly MNP-based biosensing.

## Materials and Methods

### Chemicals

Iron­(III) acetylacetonate (Fe­(acac)_3_, 99.9% trace metal basis), cobalt­(II) acetylacetonate (Co­(acac)_2_, ≥99.0%), dibenzyl ether (DBE, ≥98%), 1-octadecene (ODE, 90%), and oleic acid (OA, 90%) were purchased from Sigma-Aldrich. Sodium oleate (NaOL, >97%) was purchased from TCI, America. Zinc­(II) acetylacetonate (Zn­(acac)_2_, 95%) was purchased from Merck. Ethanol, methanol, isopropanol, acetone, and chloroform with the highest purity grade were purchased from Carl Roth, Germany. All other chemicals and reagents were purchased from Sigma-Aldrich unless otherwise stated. No further purification of the chemicals was carried out prior to their use.

### Synthesis of Mixed-Metal Ferrite Magnetic Nanoparticles

The synthesis of mixed-metal ferrites followed our previously published protocol^44^ with slight modifications. In a typical synthesis, in a 50 mL three-neck round-bottom flask, 0.25 mmol (0.064 g) of cobalt­(II) acetylacetonate, 0.5 mmol (0.131 g) of zinc­(II) acetylacetonate, and 1.0 mmol (0.353 g) of iron­(III) acetylacetonate were combined with 5 mL of DBE and 5 mL of ODE. All the syntheses were done with a bottle of DBE (batch no. BCBT7116) that was purchased in 2018 and had been in our laboratory for several years. The ratios of ligands OA and NaOL added were varied to control the morphology of the resulting nanoparticles.

To synthesize octahedral nanoparticles with a mean *D*_eff_^nom.^ of 9 nm, 5.0 mmol of OA was added to the nominal ratio of metal acetylacetonates (4.5 mmol). For tetrahedron-shaped MNPs, we introduced 1.0 mmol of NaOL with 4.0 mmol OA in the system. Increasing the NaOL concentration to 1.5 mmol, while decreasing OA to 3.5 mmol, resulted in truncated tetrahedrons with a *D*_eff_^nom.^ of 33.3 nm. Similarly, large truncated tetrahedrons with a *D*_eff_^nom.^ of 37 nm were obtained with 2.0 mmol of NaOL and 3.0 mmol of OA. Finally, extremely truncated rod-like morphologies were synthesized using an equal ratio of 2.5 mmol of NaOL and 2.5 mmol of OA.

The round-bottom flask was equipped with a 10 mm cylindrical magnetic stirring bead, a thermocouple, and a reflux condenser (with length of 13.2 mm) connected to a Schlenk line and an oil bubbler through a vacuum transfer adapter with two stopcocks. To ensure better homogeneity and degassing, the mixture was heated to 90 °C at a heating ramp rate of 4.2–4.5 °C/min and maintained for 65 min while stirring at 200 rpm. During the initial phase of degassing at low temperature, N_2_ was purged twice. By the end of degassing, the vacuum pressure reached 60–70 μbar. The stirring speed was set to 1400 rpm when no bubbling was seen. Afterward, the flask was purged with N_2_ and kept under N_2_ bed throughout the whole synthesis. The mixture was then first heated up to 130 °C at a heating ramp rate of 4.4 °C/min and held for 5 min. Subsequently, a slower heating ramp rate of ∼ 3 °C/min was applied to reach to 290 °C, and the mixture was allowed to stay at 290 °C for an additional 30 min.

The flask was cooled down to 40 °C under ambient conditions. A dark brown viscous mixture was obtained, which was diluted with 20 mL of chloroform. The diluted crude mixture was evenly divided into four 50 mL falcon tubes for a better workup. Each fraction was sonicated for 10 min before acetone was added to each tube to bring the total volume to 35 mL, followed by thorough mixing by shaking. To remove organic and inorganic impurities and to collect the nanoparticles, the mixture was then centrifuged at 8000 rpm for 10 min. The organic supernatant was decanted, and the precipitated particles in each falcon tube were dispersed in 5 mL of chloroform with vigorous sonication for 10 min. To further purify the particles and remove the polar organics residues, 3 mL methanol, 12 mL isopropanol, and 15 mL acetone were sequentially added into each falcon tube to make up a final volume of 35 mL followed by thorough mixing. The mixture was then centrifuged at 8000 rpm for 10 min, and the supernatant was decanted. This washing step with the polar solvent combination was repeated one more time. After three rounds of precipitation, the particles were dispersed in 5 mL chloroform with sonication for 10 min. To remove the residual polar solvents, 30 mL acetone was added into each falcon tube, vigorously mixed, and centrifuged at 8000 rpm for 10 min. Finally, nanoparticles were dispersed in 12 mL chloroform, sonicated for 30 min, and then stored at room temperature for further use and characterization.

### Synthesis of Mixed-Metal Oleate Precursors

The precursor study followed a similar protocol to the particle synthesis procedure. In a 50 mL three-neck round-bottom flask, 0.25 mmol (0.064 g) of cobalt­(II) acetylacetonate, 0.5 mmol (0.131 g) of zinc­(II) acetylacetonate, and 1.0 mmol (0.353 g) of iron­(III) acetylacetonate were combined with 5 mL of DBE and 5 mL of ODE. For the synthesis of the CFZ2 precursor, 1.0 mmol of NaOL and 4.0 mmol of OA were added to the synthesis mixture. For the CFZ5 precursor, 2.5 mmol of NaOL and 2.5 mmol of OA were used. The round-bottom flask was equipped with a 10 mm cylindrical magnetic stirring bead, a thermocouple, and a reflux condenser (with length of 13.2 mm) connected to a Schlenk line and an oil bubbler through a vacuum transfer adapter with two stopcocks. The mixture was degassed at 90 °C at a heating ramp rate of 4.2–4.5 °C/min and maintained for 65 min while stirring at 200 rpm. During the initial phase of degassing at low temperature, N_2_ was purged twice. By the end of degassing, the vacuum pressure reached 60–70 μbar. The stirring speed was set to 1400 rpm, when no bubbling was seen. Afterward, the flask was purged with N_2_ and kept under N_2_ bed throughout the whole procedure. The mixture was then first heated up to 130 °C at a heating ramp rate of 4.4 °C/min and held for 5 min. Subsequently, a slower heating ramp rate of 2.96 °C/min was applied to reach 150 °C, and the mixture was held at this temperature for an hour. After completion of reaction, the mixture was cooled to 0 °C and then centrifuged twice with acetone at 0 °C, 4500 rpm, for 10 min to remove organic solvents. The supernatant was discarded, and the crude product was dispersed in hexane. To further purify the precursor, solvent extraction was performed twice with ethanol and D.I water. Hexane was then removed under high vacuum in a rotary evaporator, and the precursor was dried overnight in an oven at 65–70 °C. Finally, the purified viscous precursors were stored at room temperatures for further characterizations.

### Synthesis of Zinc Oleate Precursors

The zinc-oleate precursors were synthesized following the same protocol as for the mixed-metal oleate precursors, including only 0.5 mmol (0.131g) of zinc­(II) acetylacetonate. Zinc oleate1 was synthesized with 1.0 mmol of NaOL and 4.0 mmol of OA, and zinc oleate2 was synthesized with 2.5 mmol of NaOL and 2.5 mmol of OA, matching the ligands ratio of CFZ2 and CFZ5 precursors, respectively.

### Transmission Electron Microscopy (TEM), High-Resolution Transmission Electron Microscopy (HR-TEM), and High-Angle Annular Dark-Field Scanning Transmission Electron Microscopy (HAADF-STEM)

Transmission electron microscopy analysis was carried out on a JEOL TEM microscope operating at an accelerating voltage of 100 kV. The HR-TEM analysis was conducted on a Tecnai G2 F20 Microscope from FEI, operating at an accelerating voltage of 200 kV, at the Laboratory of Nano and Quantum Engineering (LNQE), Leibniz University Hannover. The HAADF-STEM analysis was performed on an aberration-corrected JEOL Neo-ARM 200F microscope using a primary electron energy of 200 kV, at the Laboratory for Emerging Nanometrology (LENA), Braunschweig.

The samples were prepared by drop-casting 5 μL of dilute suspension of NPs in chloroform on a 300 mesh Formvar-carbon-coated copper grid and allowing the solvent to evaporate slowly at room temperature inside the fume hood. TEM images were analyzed by using ImageJ software. The size distribution and average particle size were evaluated by measuring at least 200 nanoparticles per sample.

### Inductively Coupled Plasma Optical Emission Spectroscopy (ICP-OES)

ICP-OES was performed on a Varian (715ES) instrument to determine the concentration of the elements of interest. Briefly, 50 μL of particle suspension in chloroform and 1 mL of aqua regia (HCl: HNO_3_ at 3:1 (v/v)) was pipetted into a 10 mL volumetric flask. Next, the samples were incubated at 60 °C for 1 h to facilitate the digestion process and then left inside a fume hood overnight. The next day, the samples were diluted with Milli-Q water up to a 10 mL grading level.

### Fourier Transform Infrared Spectroscopy (FTIR)

FTIR spectroscopy was performed by using a Bruker VERTEX 70 spectrometer. The samples were prepared by drop casting a small amount of viscous precursor on the diamond crystal of the FTIR. The measurements were performed with 12 scans to obtain the spectral data.

### Thermogravimetric Analysis (TGA)

Thermal gravimetric analysis was performed with a Mettler Toledo thermal analyzer TGA/DSC 1 STARe system in the temperature range from 30 to 700 °C on a 15 mg sample, with a linear heating rate of 10 K/min under nitrogen flow of 10 mL/min.

### Magnetic Property Measurement System (MPMS)

The magnetization hysteresis loops were measured using MPMS (Quantum Design). The samples were prepared by drying a 5 mL suspension of particles in chloroform to obtain approximately 6–7 mg of dried material, which was then placed in the designated sample holder. The hysteresis loops were recorded at 298 K in magnetic fields between −7 and 7 T. The applied magnetic fields were corrected for a remanence field in the chamber and the magnets by measuring the palladium standard sample. The coercive fields were corrected accordingly. Measurement errors were obtained from combining the results of three independent measurements and error propagation.

### Powder X-ray Diffraction (XRD)

The samples were prepared by drop casting highly concentrated particle suspensions on a zero-diffraction silicon wafer (⟨100⟩, 10–20 Ω cm). The measurements were conducted on a D8 Advance Bruker diffractometer, equipped with Cu Kα (2.2 kW, ceramic isolation body, focus dimension 0.04 × 12 mm^2^, Siemens) anode operating at 40 mA and 40 kV. The patterns were recorded in a parallel beam geometry over an angular range of 2θ = 10–95° in a step size of 0.02°.

## Results and Discussion

### Tailoring Ligand-Controlled Morphological Evolution: from Octahedra to Tetrahedra and Rod-Like Morphologies

In a typical thermal decomposition synthesis in the presence of carboxylic ligands, carboxylates contribute significantly to precursor formation and also act as strong capping ligands, influencing nanoparticle surface energies and directing facet-selective growth. To understand these roles in the precursor formation and growth kinetics, here, we focused on two key questions: (1) to what extent is NaOL’s role in particle formation determined by its involvement in the precursor formation and (2) how does its surface capping ability and affinity further dictate nanoparticle growth and shape? To address these questions, we have built upon our previous procedure for the synthesis of cobalt- and zinc-doped mixed metal ferrites (CFZ-MNPs)^44^ and designed a set of syntheses in which we have systematically varied the ratio of NaOL to OA from 0 to 1 while keeping the total amount of ligands to 5 mmol. We observed major changes in particle morphologies from octahedron to extremely truncated rod-like morphologies across this range.

Octahedron-shaped MNPs with rounded edges were obtained in the presence of OA only (CFZ1; Figure 1). The size analysis was performed by measuring the particle edge length along the [220] direction and converting it into the effective nominal particle size (*D*_eff_^nom.^), taking particle shape into consideration, with histograms generated by counting more than 100 particles for each sample (more details in the Supporting Information (SI), Figures S1 and S2). The octahedral MNPs exhibit a narrow size distribution, with a mean *D*_eff_^nom.^ of 9 nm ± 0.9 (*N* = 400). High-resolution transmission electron microscopy (HRTEM) analyses reveal the single-crystalline nature of these MNPs. When viewed along the [11̅0] zone axis, it can be discerned that the octahedral particles are formed by the {111} family of crystalline planes. The interplanar spacing of 2.9 and 4.8 Å can be assigned to the interplanar spacing of (220) and (111) planes of Co_*x*_Zn_*y*_Fe_3‑(*x*+*y*)_O_4_ in a cubic crystal structure, respectively (Figure [Fig fig1]1a,b). The 2D projections of octahedral MNPs, appearing cubic, rhombohedral, or hexagonal along the [100], [110], and [111] viewing directions, respectively, are shown alongside the corresponding HRTEM images (Figure S3a). These results are consistent with those reported in the literature.^28,45,46^ The addition of NaOL as a secondary ligand (molar ratio of 1:4 of NaOL: OA) to the synthesis results in truncated tetrahedron-shaped MNPs (CFZ2-Figure 1) with *D*_eff_^nom.^ of 19 nm ± 2.4 (*N* = 150). HRTEM images show two lattice fringes with interplanar spacing of approximately 2.9 Å and 4.8 Å, which can be assigned to the interplanar spacing of (220) and (111) planes of the mixed metal ferrite, along the [11̅0] zone axis, respectively (Figure [Fig fig1]2a,b).^28,36^ Increasing the molar ratio of NaOL:OA further to 1.5:3.5 results in truncated tetrahedral MNPs (CFZ3-Figure 1) with a *D*_eff_^nom.^ of 33.3 nm ± 8.6 (*N* = 100). When the ratio is set to 2:3, MNPs evolve into large truncated tetrahedral structures (CFZ4-Figure 1) with an increased *D*_eff_^norm.^ of 37 nm ± 11 (*N* = 105). The zoomed-in HRTEM image (Figure 1(4b)) displays a crossed lattice fringe with an interplanar spacing of 2.9 Å, corresponding to the (220) planes along the [11̅0 zone axis. The edge truncation and elongation in one direction give the truncated tetrahedron a distinct morphology. The 2D projections of the tetrahedron and truncated tetrahedron along different crystallographic directions are shown in Figure S3b,c.

**Figure 1:**
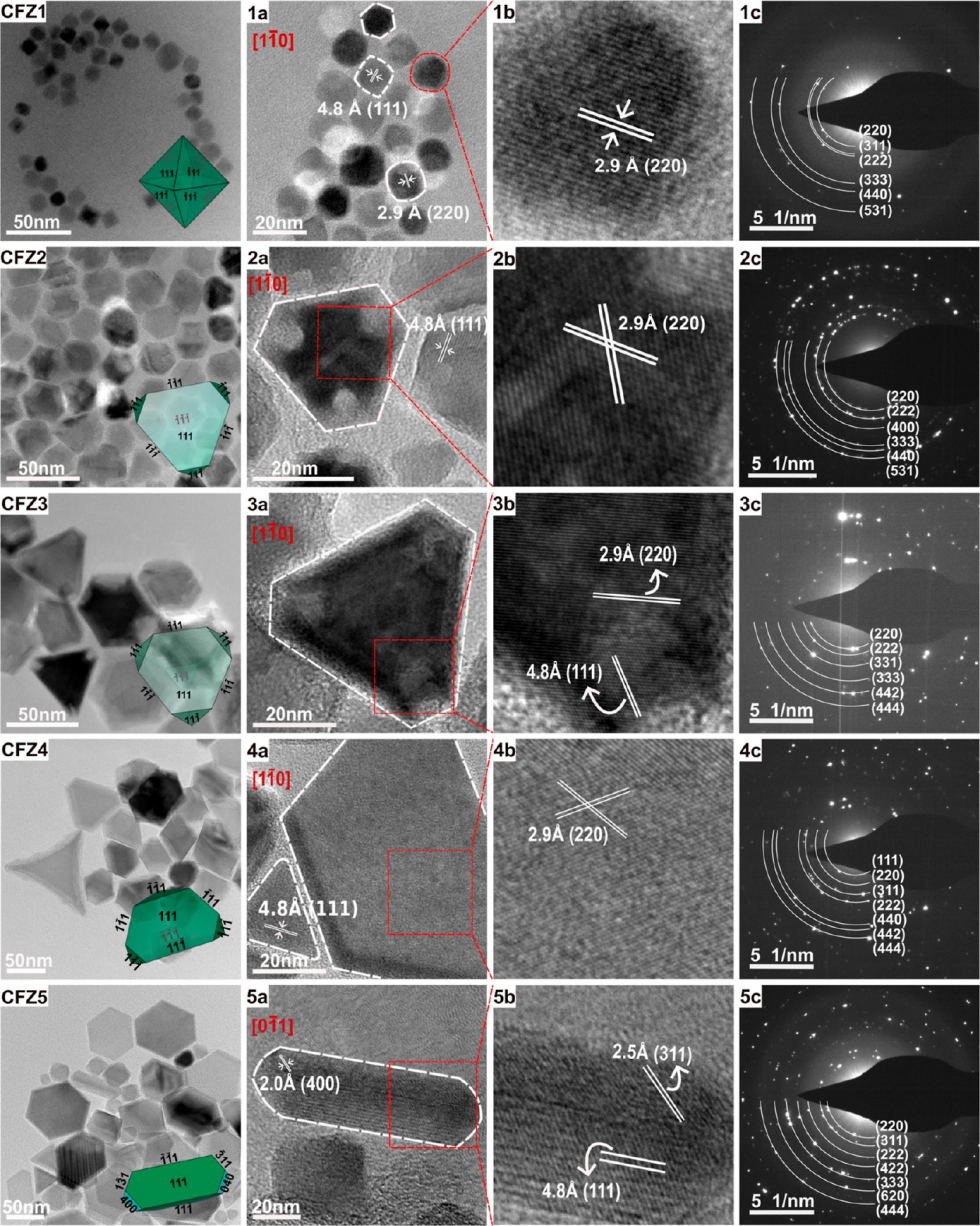
High-resolution transmission electron microscopy (HRTEM) analysis of MNPs. HRTEM images show monodisperse octahedral MNPs (CFZ1), slightly truncated tetrahedral MNPs (CFZ2), truncated tetrahedral MNPs (CFZ3), large truncated tetrahedral MNPs (CFZ4) with unique edge truncation and elongation, and a mixture of tetrahedra, nanodiscs, and rod-like MNPs (CFZ5). The insets show 3D schematic morphologies generated using Vesta crystallographic software by truncating specific planes to a certain level. For CFZ5 and for the sake of clarity, only rod-like MNPs are shown. (1a–5a) Single-particle HRTEM images. (1b–5b) The corresponding zoomed-in high-resolution images of single particles clearly exhibit distinct lattice fringes. Interplanar distances of 2.9, 4.8, 2.5, and 2.0 Å are assigned to the (220), (111), (311), and (400) planes of the CFZ MNPs (Co_*x*_Zn_*y*_Fe_3‑(*x*+*y*)_O_4_), respectively. Zone axes are written in red. (1c–5c) Selected area electron diffraction (SAED) patterns recorded from ensemble of nanoparticles for each sample, exhibiting diffraction spots corresponding to the cubic crystal structure.

Finally, when the molar ratio of NaOL to OA is equal at 2.5:2.5, a polydisperse sample consisting of a mixture of tetrahedrons, nanodiscs, and rod-shaped MNPs was obtained (CFZ5-Figure 1). The HRTEM image shows an exemplary rod-shaped MNP (Figure 1(5a,5b)) enclosed by three crystalline planes (400), (311), and (111) with interplanar spacings of 2.0, 2.5, and 4.8 Å, respectively, when observed along the [01̅1] zone axis. Since the CFZ5 sample exhibits a broad morphology distribution, average particle sizes were determined directly from the apparent edge lengths (AEL) measured from transmission electron microscopy (TEM) images and not converted to *D*_eff_^nom.^. The particle size and morphology distribution (Figure S2), determined from a total of 100 counted MNPs, indicate that tetrahedra MNPs dominate the sample population (∼60%), followed by nanodiscs (∼23%) and nanorods (16%). The average sizes of the tetrahedral and nanodisc MNPs are 24 ± 19, and 61 ± 8.3, respectively, where the latter corresponds to the diagonal length measured for nanodiscs. Two characteristic lengths were measured for the nanorods: a short length of 19 ± 3.4 and a long length of 53 ± 3. The selected area electron diffraction (SAED) patterns of all samples (CFZ1–CFZ5) consisting of octahedral, truncated tetrahedral, and nanorods (Figure 1(1c–5c)) show diffraction spots and for diffraction rings, consistent with the distinct (220), (311), (400), and (440) X-ray diffraction peaks of a cubic crystal structure.

Next, we analyzed the HRTEM images by Fourier Transform (FFT) to gain insights into lattice periodicity, crystal coherence length, dislocations, and particle’s crystalline nature, being monocrystalline or polycrystalline. The FFT analyses were performed on specific regions of the images (outlined by white rectangles in Figure 2). For CFZ1–CFZ3 samples, where the analyzed area covers nearly the whole particle, seeing characteristic diffraction spots indicate that the analyzed particles are single crystalline. In order to see the crystal arrangement of the observed planes, inverse FFT was applied to reconstruct the particle lattice image. FFT analysis for octahedral particles shows distinct bright spots corresponding to the (220) planes (CFZ1, Figure 2). This sample shows perfect lattice periodicity on the selected planes. The FFT pattern of CFZ2 exhibits diffraction spots indexed to the (220) planes. However, the inverse FFT reconstruction indicates the lack of complete lattice periodicity throughout the particle. Screw dislocations with a characteristic sheared lattice distortion (marked with red lines, Figure 2) and some planar defects are to be seen.

**Figure 2:**
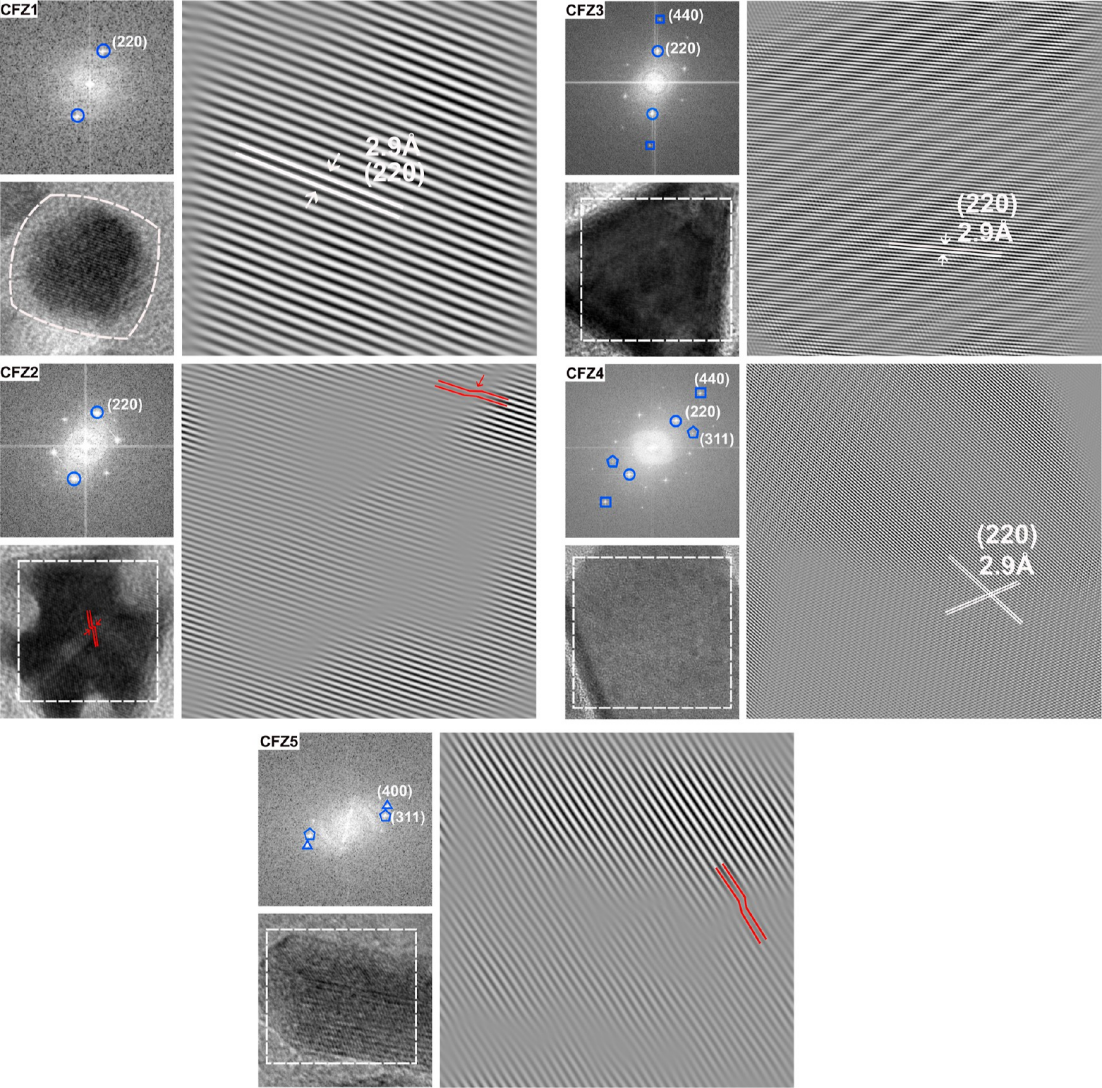
HRTEM images, corresponding fast Fourier transform (FFT), and the reconstructed inverse FFT analysis of all samples shown in Figure 1. The FFT analysis were performed on specific regions of the HRTEM images (highlighted by white squares). The FFT patterns of all samples display indexed diffraction spots for respective lattice planes. The inverse FFT images were reconstructed from the (220) reflections for CFZ1, CFZ2, CFZ3, and CFZ4 and from the (400) reflections for CFZ5. Red lines highlight regions exhibiting lattice defects in the inverse FFT images of CFZ2 and CFZ5.

The FFT pattern of CFZ3 exhibits bright spots corresponding to both (220) and (440) planes, whereas CFZ4 shows reflections from (220), (311), and (440) planes (CFZ3 and CFZ4, Figure 2). The corresponding inverse FFT images were reconstructed after excluding the contributions of (440) reflections in CFZ3 and (311) and (440) reflections in CFZ4, revealing well-ordered and continuous lattice fringes with no visible dislocations or discontinuities in the select areas. Our HRTEM and FFT investigations cannot fully exclude dislocations and defects in these two samples. The FFT pattern of rod-like NPs shows reflections from the (311) and (400) planes (CFZ5, Figure 2). The inverse FFT was reconstructed by masking the reflections from the (311) planes, thereby isolating the respective lattice fringes associated with each set of planes. Similar to CFZ2 particles, here, we observe a mixture of screw dislocations and planar defects (highlighted by red lines in the inverse FFT image). The HRTEM and corresponding inverse FFT images thus reveal localized lattice distortions within particles in CFZ2 and CFZ5 particles, representing local lattice strain. Nevertheless, the overall uniformity of lattice fringes and distinct diffraction spots in the FFT patterns, yet taking limited sampling area into consideration, strongly indicates that all synthesized nanoparticles are single crystalline.

To understand how the morphological and structural properties of particles are correlated, we performed powder X-ray diffraction analysis (Figure 3). All reflections can be assigned to a cubic crystal structure for all the samples, indicating that all synthesized MNPs are single-phase with no detectable signal from other crystalline phases. The peak positions of all samples match, indicating a comparable doping level of Zn and Co into the structure and agreeing well with the ICP results (Table 1). We further elaborated on the XRD results by doing single peak analysis to correlate the effective crystallite size (*D*_eff_) from XRD with the nominal particle size (*D*_eff_^nom.^) from TEM investigations. We focused on the (220) diffraction line, as it is characteristic of the spinel structure, showing cation ordering in tetrahedral sites.^47^ The *D*_eff_ was calculated along the normal of the (220) diffraction line using the Scherrer equation, *D*_eff_ = (*k*_hkl_λ)/(2ω cos­(θ)), with *k* the Scherrer constant, λ = 0.154 the X-ray wavelength in nm, and 2ω and θ the full width at half-maximum (fwhm) and the Bragg angle in radian. We considered the particle shape anisotropy in calculations by considering different *k* values for different shapes: for octahedron, *k*_220_ = 0.810 and for tetrahedron, *k*_220_ = 0.747 ^48^, to estimate *D*_eff_ more accurately. We also took the effect of particle shape into account by defining a shape-independent size metric, which is the edge length *L* of a cube that is circumscribed to a polyhedron (see Figure S1 for more details). For an octahedron, the nominal crystallite size (*D*_eff_^nom.^) along the [220] crystallographic direction is 0.530 × *L*. For a tetrahedron, the *D*_eff_^nom.^ = 0.707 × *L* along the same direction.^48^ The *D*_eff_ versus *D*_eff_^nom.^ plot reveals some interesting features (Figure 3b). For the octahedron-shaped MNPs (CFZ1), *D*_eff_ and *D*_eff_^nom.^ match well, indicating perfect crystal ordering and periodicity throughout the whole crystal (Figure 3b). This result agrees with the inverse FFT analysis, where well-ordered lattice fringes were observed. However, upon transforming from an octahedron to a tetrahedron and while increasing *D*_eff_^nom.^ from 9 to 19 nm, *D*_eff_ drops even slightly. This result suggests that the lattice ordering along the [220] direction is not coherent in CFZ2 particles. This was seen as distorted lattice periodicity due to linear and planar defects in the reverse FFT (Figure 2), leading to a fairly large discrepancy (∼75%) between *D*_eff_^nom.^ and *D*_eff_ in this sample. Interestingly, upon forming larger and more truncated tetrahedral particles by going to CFZ3 particles, *D*_eff_^nom^ increases for ∼75% while *D*_eff_ enlarges for ∼57%. The fact that two sizes differ less is intriguing and may well indicate a better lattice periodicity, as also seen in our FFT investigations (Figure 2). By forming even larger particles in CFZ4 and CFZ5 samples, *D*_eff_ changes marginally, suggesting that the strong truncation of {111} vertices and thus formation of nanodiscs and rod-like MNPs does not allow the crystallite size along [220] to increase alongside the particle physical size. In fact, defining a proper particle physical size for such highly truncated tetrahedra, rod-like NPs and nanodiscs is highly challenging. To shed light on this issue, we plotted *D*_eff_ as a function of AEL (the particle size along the (220) direction, Figure 3c). Overall, we observe that discrepancies between XRD and TEM sizes are reduced. For CFZ5 particles, by considering the size along the shortest length of rod-like MNPs and nanodiscs, a very good agreement between *D*_eff_ and the particle physical size can be seen. All in all, we hypothesize that a combination of two effects leads to large discrepancies between sizes from XRD and TEM. First, dislocations and defects breaking the periodicity of the lattice along the [220] direction contribute to some extent. Second, truncation of {111} vertices along the [220] direction, becoming more significant by having more NaOL, keeps the particle’s thickness from growing together along other dimensions. For this exact reason, the discussed discrepancies are very dominant for CFZ5 particles, where the truncation is extended toward 2D nanodiscs and rod-like NPs.

**Figure 3:**
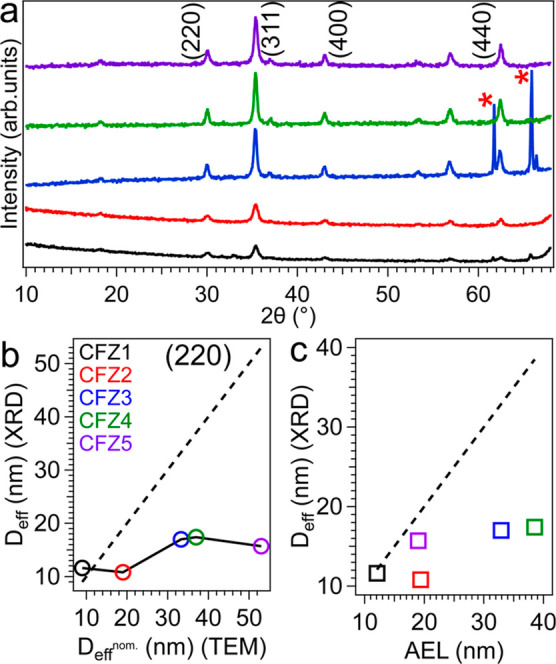
(a) Powder X-ray diffraction (XRD) pattern of all particles shown in Figure 1. Reflections highlighted by red stars are from the silicon substrate. (b) The effective particle size *D*_eff_ versus the nominal effective size *D*_eff_^nom.^ along the normal of the (220) diffraction line by taking the particle shape into account. (c) *D*_eff_ from XRD versus AEL (apparent edge length). The AEL size index is a measure of particle size along the [220] direction. For the CFZ5 sample, the short length of nanodiscs and rod-like NPs along the [220] crystallographic direction is plotted (see the Supporting Information for size histograms). The dashed lines in panels (b) and (c) present an ideal situation wherein *D*_eff_ values are equal to *D*_eff_^nom.^ or AEL between these two values. Considerations on the conversion of the physical size from TEM to *D*_eff_^nom.^ are given in the SI (Figure S1). Same color coding is applied to all panels.

**Table 1:** Feed Ratio of Surfactants and Final Composition of Co, Zn, and Fe in All Synthesized MNPs, Their *D*_eff_^nom^ ± Standard Deviation (SD) (*D*_eff_^nom^ Histograms Are Shown in Figure S2), Maximum Magnetization *M*_max_ ± SD at 298 and 5 K, and Coercive Field μ_0_H_c_ ± SD at 298 and 5 K as Derived from ICP-OES, TEM, and Magnetization Hysteresis Loops Measurements and Analyses^c^

	surfactant ratio (mmol)		MNPs composition (based on ICP)	*D*_eff_^nom.^ (nm)	*M*_max_ (emu/g) 298 K	*M*_max_ (emu/g) (Fe + Co)	μ_0_H_c_ (mT) @ 298 K	*M*_max_ (emu/g) 5 K	μ_0_H_c_ (mT) @ 5 K
	NaOL	OA				298 K			
CFZ1	0	5.0	Co_0.3_ Fe_2.5_ Zn_0.2_ O_4_	9 ± 0.9	56.5 ± 1.6	84	44.1 ± 25.7	64.36 ± 0.5	1073.3 ± 0.3
CFZ2	1.0	4.0	Co_0.33_ Fe_2.29_ Zn_0.38_ O_4_	19 ± 2.4	72.4 ± 0.9	116	28.3 ± 2.0	91.8 ± 1.5	715.8 ± 9.3
CFZ3	1.5	3.5	Co_0.27_ Fe_2.3_ Zn_0.43_ O_4_	33.3 ± 8.6	86.03 ± 1.0	141	16.9 ± 0.2	117.5 ± 3	267.9 ± 0.7
CFZ4	2.0	3.0	Co_0.25_ Fe_2.45_ Zn_0.3_ O_4_	37 ± 11	68.8 ± 0.9	107	31.5 ± 0.3	84.7 ± 1.3	359.7 ± 1.3
CFZ5	2.5	2.5	Co_0.21_ Fe_2.52_ Zn_0.27_ O_4_	^a^	57.9 ± 0.1	^b^	42.2 ± 0.4	67.3 ± 0.2	496.03 ± 0.6

a CFZ5 particles have different shapes; see Figure S2 for complete particle size and morphology distribution.

b Normalization of *M*_max_ (emu/g) to the magnetic component (Fe + Co) depends on the particle size distribution. Since CFZ5 exhibits broad morphology distribution, normalizing *M*_max_ data to the magnetic component (Fe + Co) would not accurately represent the magnetic behavior of the entire sample.

c Measurement uncertainties are determined from three independent measurements.

### NaOL Lowers Precursor Decomposition Temperature and Leads to Kinetically Controlled Morphologies

The effects of the type and concentration of ligands on the morphology and size of MNPs suggests that they play a crucial role in the precursor formation and their decomposition kinetics.^16,49,50^ To address these questions and shed light on the underlying formation mechanisms of the observed morphologies, we synthesized mixed-metal oleate precursors at two different NaOL to OA ratios: CFZ2 (1.0 mmol of NaOL +4.0 mmol of OA) and CFZ5 (2.5 mmol of NaOL +2.5 mmol of OA). Among the five samples, we focused on CFZ2 and CFZ5 for detailed precursor analysis as they represent the lowest and highest NaOL concentrations, respectively, thus allowing us to understand the full spectrum of NaOL’s influence on precursor chemistry. The precursors were synthesized using the particle synthesis procedure except that the reaction was terminated after an hour of incubation at 150 °C (more details in the Method section). As a result of ligand exchange between metal-bound and added ligands, the metal oleate precursors are well formed at these conditions.^51^

To understand how the coordination chemistry of carboxylate groups with the metal ions depends on the NaOL to OA ratio, we performed attenuated total reflectance-Fourier transform infrared spectroscopy (ATR-FTIR) on purified precursors following specific purification steps. The IR spectrum of the CFZ2 precursor shows a small peak at 580 cm^–1^, which corresponds to the asymmetric stretching of the triangular Fe_3_O core (Fe–O–Fe).^12,16,52^ When one of the iron atoms is substituted with a divalent atom (M^2+^), the triangular C_3_ symmetry is broken and the point group is changed from D_3h_ to C_2v_. This symmetry breakage results in a peak splitting of the asymmetric vibration, producing two peaks at 550 and 720 cm^–1^.^16,52^ This splitting behavior is indeed observed in both the CFZ2 and CFZ5 precursors. The CFZ2 precursor shows absorption peaks in the region of 720–740 cm^–1^, whereas the CFZ5 precursor displays more pronounced peaks at the 695–730 cm^–1^ range, indicating a successful formation of Co–Fe bimetallic-oxo complexes (Figure 4a). Gupta and co-workers reported that in the synthesis of bimetallic ferrite NPs involving late transition metal ions with oxidation states of 2+, including Co^2+^, Mn^2+^, Ni^2+^, and Cu^2+^, bimetallic-oxo clusters with composition of [MFe_2_O­(oleate)_6_] are formed as precursors for Co^2+^, Mn^2+^, and Ni^2+^, except for Cu^2+^.^17^ The Hyeon group observed similar behavior with Zn^2+^, forming Zn-oleate instead of bimetallic-oxo clusters with Fe.^16^

**Figure 4:**
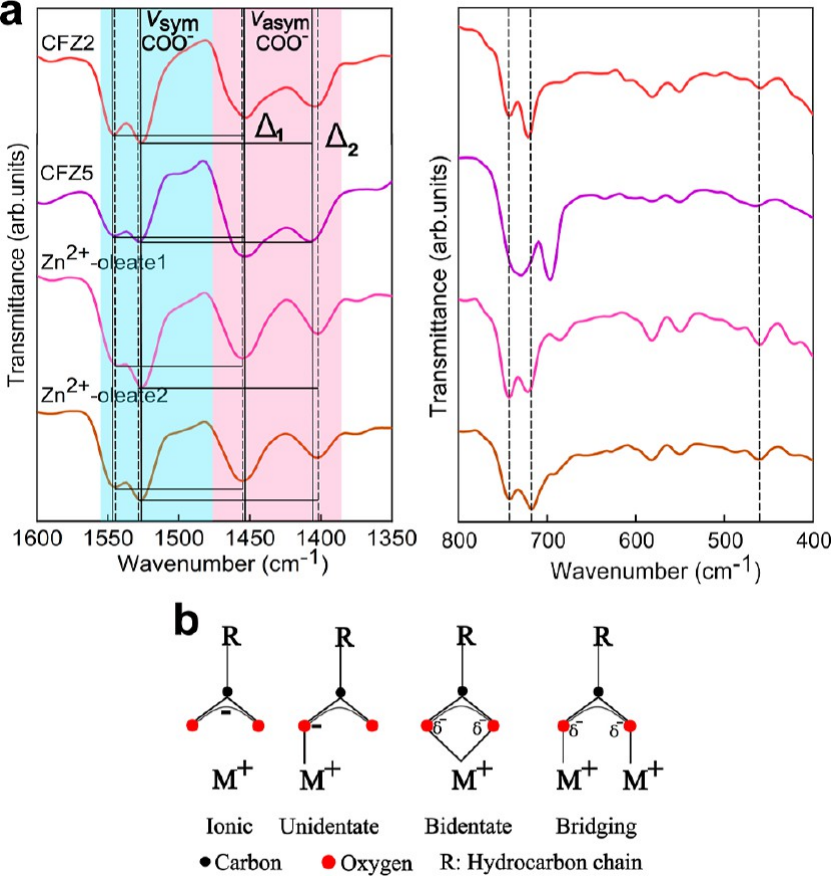
Attenuated total reflectance-Fourier transform infrared (ATR-FTIR) spectroscopy studies on mixed-metal oleate and zinc oleate precursors. (a) IR spectra of CFZ2 (1.0 mmol NaOL +4.0 mmol OA) and CFZ5 (2.5 mmol NaOL +2.5 mmol OA) precursors exhibiting characteristic peaks of MFe_2_O core and Zn^2+^ oleate1 (1.0 mmol NaOL +4.0 mmol OA) and Zn^2+^ oleate2 (2.5 mmol NaOL +2.5 mmol OA) exhibiting Zn–O broad peaks in the 450–570 cm^–1^ region. (b) Schematic drawings of four different coordination modes of metal atoms with the carboxyl group. The solid lines represent CFZ2 and CFZ5 precursors and dashed lines represent Zn^2+^ oleate1 and Zn^2+^ oleate2 precursors.

The peak for Zn–O typically appears in the range of 450–650 cm^–1^ at low temperatures,^53–55^ which is often difficult to identify. However, in our sample with 2.5 mmol of NaOL (CFZ5 precursor), a broad peak at 460 cm^–1^ suggests the presence and formation of Zn-oleate at high NaOL concentrations. To further validate this, we synthesized two zinc oleate reference samples: zinc oleate1 (1.0 mmol of NaOL +4.0 mmol of OA, pink line in Figure 4a) and zinc oleate2 (2.5 mmol of NaOL +2.5 mmol of OA, brown line in Figure 4a) matching the ligand ratios used in CFZ2 and CFZ5 precursors, respectively. Both samples exhibited broad peaks in the 450–570 cm^–1^ region, corresponding to Zn–O stretching vibrations, along with small peaks close to 700 cm^–1^ indicative of the rocking vibrations of long CH_2_ alkyl chains. However, unlike the CFZ2 and CFZ5 precursors, the peaks observed near 700 cm^–1^ for zinc oleate1 and zinc oleate2 did not exhibit significant splitting or intensity enhancement. These findings confirm that ligand composition directly influences Zn-oleate formation, and the spectral features observed in CFZ2 and CFZ5 precursors presumably result from bimetallic-oxo complex formation.

The IR spectra of the CFZ2 precursor resemble closely that of the CFZ5 precursor in the region between 1400 and 1600 cm^–1^, corresponding to symmetric and asymmetric stretching of metal carboxylates.^56–58^ The zinc oleate precursors exhibit distinct differences in this range. Carboxylate groups can interact with metal cations in four different configurations: ionic, unidentate, bidentate, and bridging^27,59^ (Figure 4b). The difference between the asymmetric and symmetric (COO^–^) bands positions (Δ = ν_asym_ – ν_sym_) helps infer the coordination mode.^30^ A Δ < 110 cm^–1^ refers to a bidentate coordination, a Δ > 200 cm^–1^ indicates a unidentate coordination, and for intermediate values (110 cm^–1^ < Δ < 200 cm^–1^), a bridging mode is expected.^30,57,58^ To calculate Δ values, the band splitting has been considered, and the minimum Δ_1_ and maximum Δ_2_ values have been obtained, which are indicative of both bidentate (∼92 cm^–1^) and bridging (∼120 cm^–1^) coordination in both CFZ2 and CFZ5 precursors (Figure 4a). In contrast, zinc oleate1 and zinc oleate2 exhibit primarily bidentate coordination with Δ values of ∼89 and 72 cm^–1^.

Next, we tested whether differences in the formation of bimetallic and zinc oleate precursors would reflect in their thermal decomposition profiles by carrying out thermogravimetric analyses (TGA) and derivative thermogravimetric (DTG) analyses on three different precursors (Figure 5). We observed distinct decomposition profiles depending on the amount of NaOL that is fed into the synthesis. The CFZ2 precursor decomposes over one-step, beginning at ∼200 °C and completing at ∼450 °C. This one-step decomposition behavior suggests that three metal coordinating oleate groups with a similar binding energy are associated with metal ions. Interestingly, its decomposition profile closely resembles the precursor synthesized with only 5 mmol of OA for CFZ1 (black line in Figure 5). This resemblance indicates that NaOL is minimally involved in precursor formation in CFZ2 and acts predominantly as a capping ligand, passivating {111} facets and forming tetrahedral-shaped nanoparticles. As a result, nearly all metal ions in CFZ2 are coordinated with carboxylates originating from OA, leading to a uniform, one-step decomposition pattern.

**Figure 5:**
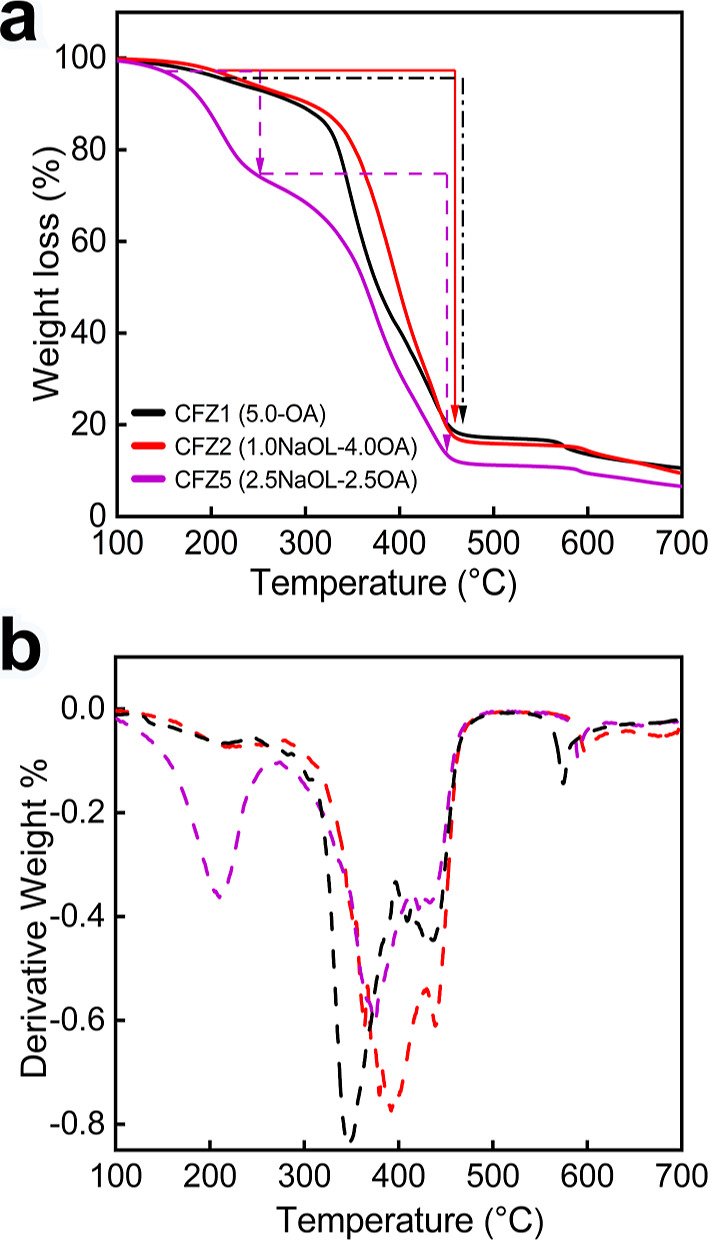
Thermal decomposition profiles of mixed-metal precursors depend on the NaOL to OA ratio. (a) TGA and (b) DTG curves of three precursors highlighting their distinct decomposition behavior. The precursor synthesized with 5.0 mmol OA (CFZ1) and 1:4 NaOL: OA (CFZ2) ratio exhibits a single-step decomposition profile, as indicated by a single major weight loss peak clearly visible in the DTG curve (panel b). Conversely, the precursor synthesized at a 2.5:2.5 NaOL:OA ratio (CFZ5) shows a two-step decomposition profile, which is more evident in the DTG curve (panel b).

In contrast, the decomposition profile of the CFZ5 precursor (formed at 2.5:2.5 mmol NaOL: OA) differs significantly, particularly at temperatures below ∼250 °C. At first glance, we observed that the CFZ5 precursor decomposes at lower temperatures, as clearly seen by its TGA curve that is shifted to lower temperatures (Figure 5a). A closer examination reveals a two-step decomposition process, with the first weight loss occurring between 150 and 250 °C and the second weight loss step between 250 and 450 °C. This two-step decomposition profile can be better discerned by looking at the derivative of weight loss where two sharp endothermic peaks are seen (Figure 5b). The decomposition profiles of zinc oleate1 and zinc oleate2 closely resemble those of CFZ2 and CFZ5, respectively (Figure S4), further supporting our hypothesis that ligand composition influences the precursor decomposition behavior.

Putting FTIR and TGA findings together, we propose the following model. In the CFZ5 precursor, there are two types of coordinating oleate groups that differ in their metal-ion binding energy. Qualitatively, one oleate group binds relatively weaker than the other two groups to metal ions. By comparing the CFZ2 and CFZ5 precursors at temperatures <250 °C, we observe an increased involvement of the relatively weak binding oleate group in the precursor by feeding more NaOL in the synthesis. In CFZ5, NaOL is involved in the formation of both Co–Fe bimetallic complexes along with OA and Zn oleate precursors, resulting in a broader distribution of binding strengths within the precursor. Our data indicate that not only the total amount of oleate ligands but also their nature plays a crucial role in the precursor chemistry and decomposition behavior.

### Mixed Ligand Coordination Promotes Early Precursor Decomposition, Altering the Nucleation Temperature and Favoring Kinetic Growth Pathways

To investigate how lowering the precursor decomposition influences the nucleation and growth pathways, we monitored the synthesis reactions of CFZ2 and CFZ5 samples by taking aliquots at different temperatures and times. For CFZ2, aliquots were collected at 250 °C, 280 °C, 290 °C (0 min), and 290 °C (2 min) as well as at the end of 30 min growth time. After the samples were washed using the same procedure as for collecting the NPs in chloroform, the samples were analyzed with transmission electron microscopy (TEM). For CFZ2, no NPs were detected at the 250 and 280 °C time point, indicating that no stable nuclei are formed at this temperature. A small number of very fine tetrahedron-shaped nanoparticles appears at 290 °C (0 min), and their morphology becomes more evident with an increase in their population at 290 °C (2 min) (CFZ2, Figure 6). These findings suggest that nucleation occurs within a narrow window between 280 and 290 °C. Time-lapse synthesis was performed in a similar manner for CFZ5, with samples collected at the same temperature intervals. In contrast to CFZ2, the formation of perfectly shaped tetrahedral MNPs was already evident at 280 °C in CFZ5 (inset), with more defined morphologies emerging at 290 °C (0 min). By the end of the 30 min growth period, the particles were fully formed, indicating that growth had completed (CFZ5, Figure 6).

**Figure 6:**
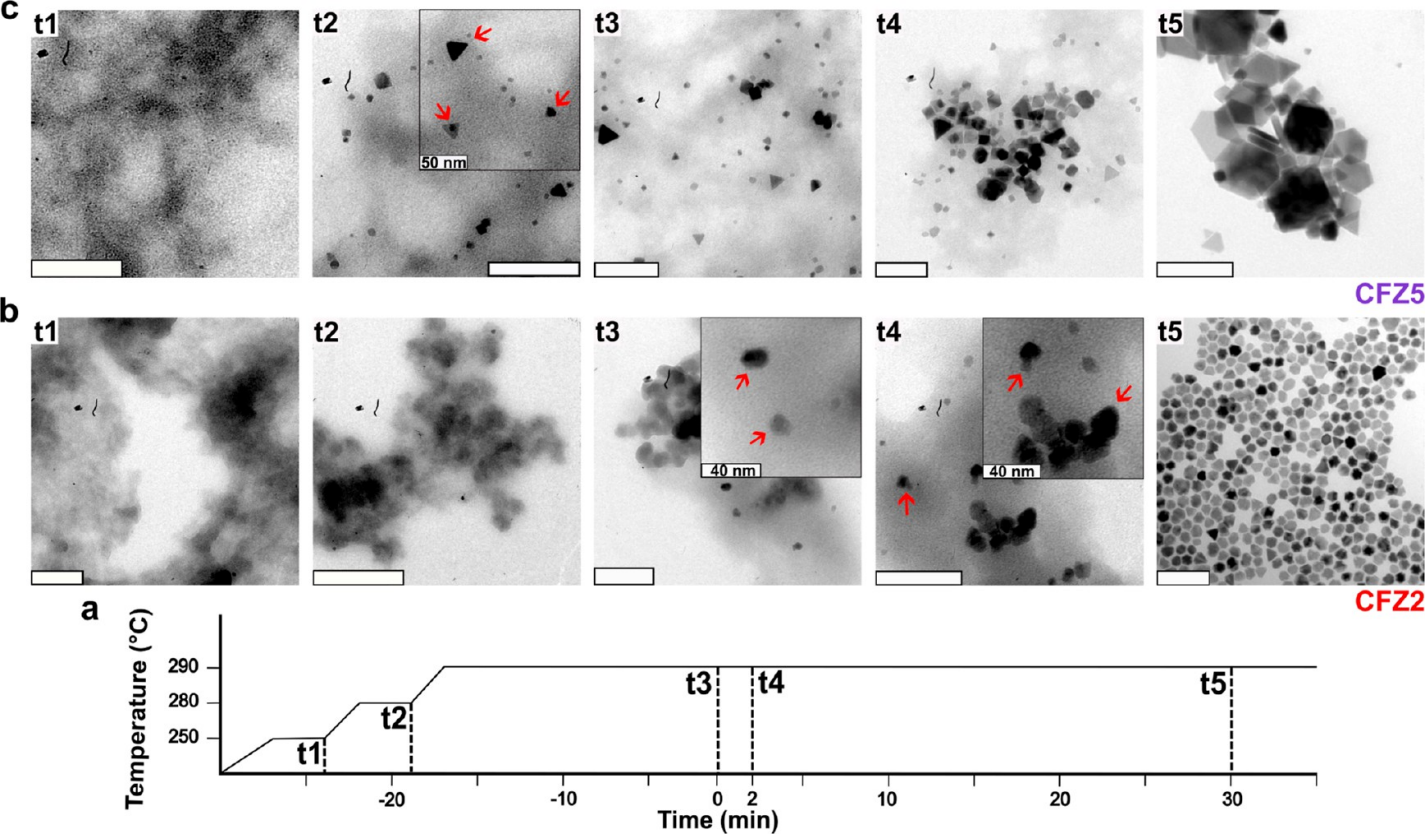
Transmission electron microscopy (TEM)­studies on aliquots taken at different temperatures and times. TEM images of nanoparticles obtained at (panel a) specific reaction time points and also at different growth times for (panel b) CFZ2 and (panel c) CFZ5. The scale bars correspond to 100 nm, unless otherwise stated. The insets show enlarged TEM images for improved clarity of the tetrahedral morphology of the formed crystallites.

These results suggest that low NaOL concentrations, as in the case of CFZ2, lead to delayed nucleation. In CFZ2, NaOL is minimally involved in precursor formation, and nearly all metal ions are coordinated to oleate ligands with similar binding energies. This yields a more uniform precursor that undergoes a single-step decomposition, as evidenced by the TGA profile of CFZ2 (red line in Figure 5). Conversely, the higher NaOL concentration in CFZ5 promotes the formation of mixed oleate complexes. This leads to early precursor decomposition, lowering the nucleation temperature and shifting the growth toward kinetically controlled pathways.

### Precursors with Mixed Coordinating Oleates Lead to Extreme and Stochastic Passivation and Truncation of {111} Planes by NaOL

Considering all of our findings together, we propose two particle growth pathways that depend on the availability and the nature of the oleates in the synthesis. When a close to nominal amount of OA is available in the synthesis (nominal = 4.5 mmol, corresponding to a 1:1 molar ratio with the total amount of all three metal acetylacetonates, based on their feed ratio in synthesis), OA acts as the primary ligand providing an oleate group to all three metal ions. As indicated by the TGA data of CFZ1 and CFZ2, the decomposition of OA begins with a single decomposition step starting at ∼160 to 200 °C.^60^ This temperature window aligns well with the formation of stable metal oxide precursors in which Co^2+^ and Fe^3+^ forms bimetallic oxo-metal complexes with six oleate ligands in bridging conditions, whereas Zn^2+^ forms mononuclear zinc oleate, as evidenced by our results and previous studies^16,17^ (Figure 7a). These precursors decompose and upon reaching higher temperatures transform into monomers,^6^ which then accumulate and supersaturate, triggering a burst nucleation event.^61^ In the cubic close-packed face-centered crystal (FCC) structure, the surface energies of low-index facets follow the order from lowest to highest as {111} < {100} < {110}.^62^ The {111} facets are the most densely packed with the lowest chemical potential and lowest reactivity, whereas the {100} facets are the least densely packed with the highest relative chemical potential and highest reactivity.^27,62^ As a result, the system prefers to form thermodynamically stable octahedral MNPs to lower the total energy. The morphology of initial crystals is also governed by the order of chemical potential of low-index facets following μ_m_ > μ_{100}_ > μ _{110}_ > μ_{111}_, where μ_m_ is the chemical potential of monomers.^7,62,63^ The chemical potential of monomers is defined by

1 $$\mu_{m}=\mu_{m}^{0}+RT\ln\left[\frac{C_{m}.\gamma_{m}}{C_{0}}\right]$$

where μ_m_^0^ is the chemical potential of monomers in a reference state and is a constant, *C*_m_ is the concentration of monomers, *C*_0_ is the concentration in reference state, and γ_m_ is the activity coefficient of monomers in solution.^7^ The rate at which monomers are deposited on each facet is determined by the difference in the chemical potential between monomers and crystal facets (Δ*E*_m,i,{*hkl*}_), specific activation energy barrier, that is given by the Arrhenius equation

2 $$k_{m,i,\left\{hkl\right\}}=Ae^{-\Delta Em,i,\left\{hkl\right\}/RT}$$

where *k*_m,i,{*hkl*}_ is the rate constant of monomer deposition *i* on {*hkl*} planes; *A* is the pre-exponential factor; Δ*E*_m,i,{*hkl*}_ is the activation energy barrier for monomer deposition *i* on {*hkl*} planes; *R* is the universal gas constant; and *T* is the temperature. Since all facets are exposed to the same monomers, deposition occurs simultaneously on all facets at different rates, determined by their rate constants *k*_m,*i*, and {*hkl*}_. Due to the dense atomic packing of {111} facets, they are the least reactive and hence exhibit the highest energy barrier, leading to greater steric hindrance for monomer deposition and the slowest growth. In contrast, the more reactive {110} and {100} facets have lower energy barriers, allowing monomers to deposit more readily and resulting in faster growth. Consequently, the {110} and {100} facets grow out and eventually diminish, whereas the stable {111} facets are retained in primary particles.^7,62^ This selective growth leads to the formation of homogeneous, thermodynamically stable monodisperse octahedrons enclosed by eight {111} facets to minimize the surface energy (CFZ1, Figure 7b).

**Figure 7:**
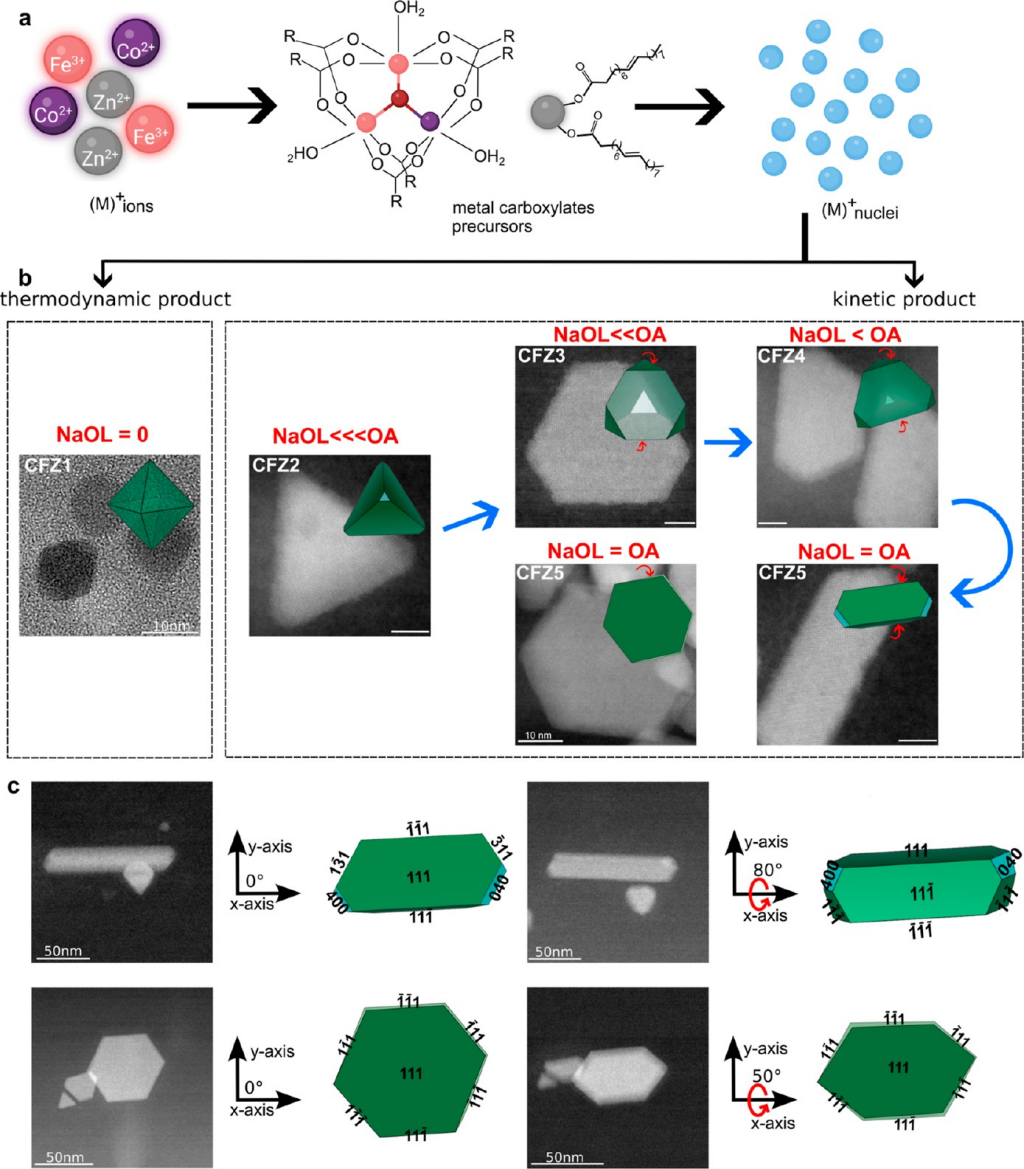
Growth mechanism of kinetically driven morphologies of mixed-metal ferrites. (a) Formation of bimetallic oxo-metal complexes and mononuclear zinc oleate as precursors. (b) HRTEM and high-angle annular dark-field scanning transmission electron microscopy (HAADF-STEM) images along with the schematic illustration of final morphologies showing the effect of increasing NaOL concentration. Initially, in the absence of NaOL, thermodynamically controlled octahedra with well-defined {111} facets are formed. As NaOL concentration increases, strong truncation (red arrows) of {111} facets by NaOL leads to kinetically controlled morphologies, whereas blue arrow indicates the evolution of morphologies. Scale bars are 5 nm unless otherwise stated. (c) HAADF-STEM images and corresponding 2D projections of rod and disc-shaped MNP at 0° (left) and 50° (right) tilt around the *x*-axis, highlighting changes in projection and shadowing. While the images were acquired at a 50° tilt (right), the schematic representation for nanorods is shown at a higher tilt angle of 80° to better match the observed particle morphology and orientation in the HAADF-STEM image. 3D morphologies were generated using VESTA crystallography software by selectively truncating certain crystallographic planes to defined extents.

The nucleation and growth processes go through very different pathways when NaOL is present. We obtained kinetic morphologies by increasing NaOL and reducing the OA concentration, as the system seems not to follow the discussed energy and chemical potential minimization.

The addition of NaOL in the synthesis served two purposes: as an additional source of ligands by providing oleate groups to metal ions to form precursors and also as a capping ligand. NaOL’s role in precursor formation is less pronounced in CFZ2 (1.0 mmol NaOL), forming zinc oleate at higher temperatures, thus leading to higher nucleation temperatures of 280–290 °C, evidenced by our time lapse studies of CFZ2 (time point t3, Figure 6). The thermal decomposition profiles of zinc oleates (Figure S4) provide indirect evidence of their stability, showing that zinc oleate decomposes at temperatures higher than those of bimetallic precursors. Initial stable crystallites have a tetrahedron morphology, regardless of the amount of NaOL added, as is clearly seen from the time-lapse studies (Figure 6). This means that only {111} facets are exposed on those crystallites. At lower NaOL concentration (CFZ2), it acts primarily as a capping ligand regulating the surface energy of {111} facets, likely due to selective binding of OL^–^ ligands to metal ions on the plane forming tetrahedron-shaped MNPs with slight truncation at some vertices (CFZ2, Figures 7b, 1, and 2a).^28^ Different {111} planes may have different levels of exposure at very initial stages of growth, thus being passivated with NaOL to different extent. In other words, the main {111} facets forming a tetrahedron particle are more densely passivated than the facets at the vertices, causing some of these {111} facets to survive and some to vanish rather stochastically. The extent of truncation becomes more extreme as the amount of NaOL increases. Increasing the NaOL concentration increases its availability as a capping ligand, which in turn increases the passivation of {111} facets, predominantly at the vertices, thereby resulting in the selective and often nonuniform truncation of {111} facets at the vertices. This stochastic truncation leads to largely truncated tetrahedrons, with unique edge truncation and elongation in a specific direction, giving nanoparticles a distinct morphology (CFZ3 and CFZ4, Figures 7b and 1(3a,4a)).

The truncation of these facets becomes more extreme with increasing NaOL further. At 2.5 mmol of NaOL, a polydisperse mixture of tetrahedrons and disc-shaped and rod-like morphologies are seen (CFZ5, Figures 7b and 1). The formation of rod-like MNPs at this concentration of NaOL is due to the strong passivation of at least two (111) facets at the vertices. We probed a single rod-shaped MNP using high-angle annular dark-field scanning transmission electron microscopy (HAADF-STEM) and found that indeed the three-dimensional shape of this peculiar morphology is the product of extreme truncation of the {111} planes (Figure 7c). We further confirmed the morphology of the large flat-looking particles in CFZ5 by acquiring HAADF-STEM images at a 50° degree tilt around *x*-axis, revealing their platelet-like/nanodisc structure. The high-resolution HAADF-STEM images for samples (CFZ2-CFZ5) are shown in Figure S5, again demonstrating high crystalline quality and lattice ordering in tetrahedral and truncated tetrahedral particles. Our HAADF-STEM images clearly show that these particles are single crystalline.

To validate our proposed mechanism for kinetically controlled morphologies by tuning the molar ratios of NaOL and OA, we synthesized two control samples. In the first experiment, we reduced the OA concentration in CFZ1 to 2.5 mmol to determine whether the reduced OA concentrations lead to kinetically controlled rather than thermodynamically controlled growth pathways, while keeping all other conditions constant. In this sample, octahedral MNPs with an average diameter of 48 nm are obtained, as revealed by HRTEM analysis (Figure 8a). The MNPs exhibit rhombus-shaped projection when viewed along the [11̅0] zone axis, which is consistent with the projection of an octahedron.^46,64–66^ The octahedral morphology of synthesized MNPs can be inferred from the visible (111) and (220) lattice planes of the cubic crystal structure. The measured interfacial angle of 74° between two (111) facets, which is obtained by extending the projected facet edges, is in good agreement with the nominal angle of 70.5°. These observations support our hypothesis that OA shows equal affinity to all low-index facets, binding to all crystal facets and promoting uniform monomer deposition. Due to the higher activation barrier for growth on {111} facets relative to {100} and {110}, monomer deposition on {111} facets is the slowest, leading to their passivation and the formation of octahedral nanoparticles enclosed by eight {111} facets to minimize surface energy. To understand the role of NaOL in the formation of tetrahedra, we performed the second control experiment by adding 1.0 mmol of NaOL to 2.5 mmol OA (Figure 8b). Tetrahedron-shaped MNPs are formed by this modification, as confirmed by the tilt-image of the HRTEM analysis, which revealed their three-dimensional geometry. This finding further supports our claim that NaOL is essential for promoting tetrahedral morphologies. These control experiments yielded three key findings that support our particle growth pathways. First, the nucleation and growth pathways of our MNPs are robust, since reduction of OA from 5 mmol to 2.5 still results in octahedral MNPs. Second, the reaction goes through thermodynamically controlled processes forming octahedral MNPs when NaOL is absent (Figure 8a). Third, the combination of NaOL with reduced OA is necessary to achieve a kinetic morphology. NaOL plays a crucial role in the formation of precursors and also acts as a ligand that regulates the passivation of {111} facets, leading to their dominance in the final nanoparticle morphology.

**Figure 8:**
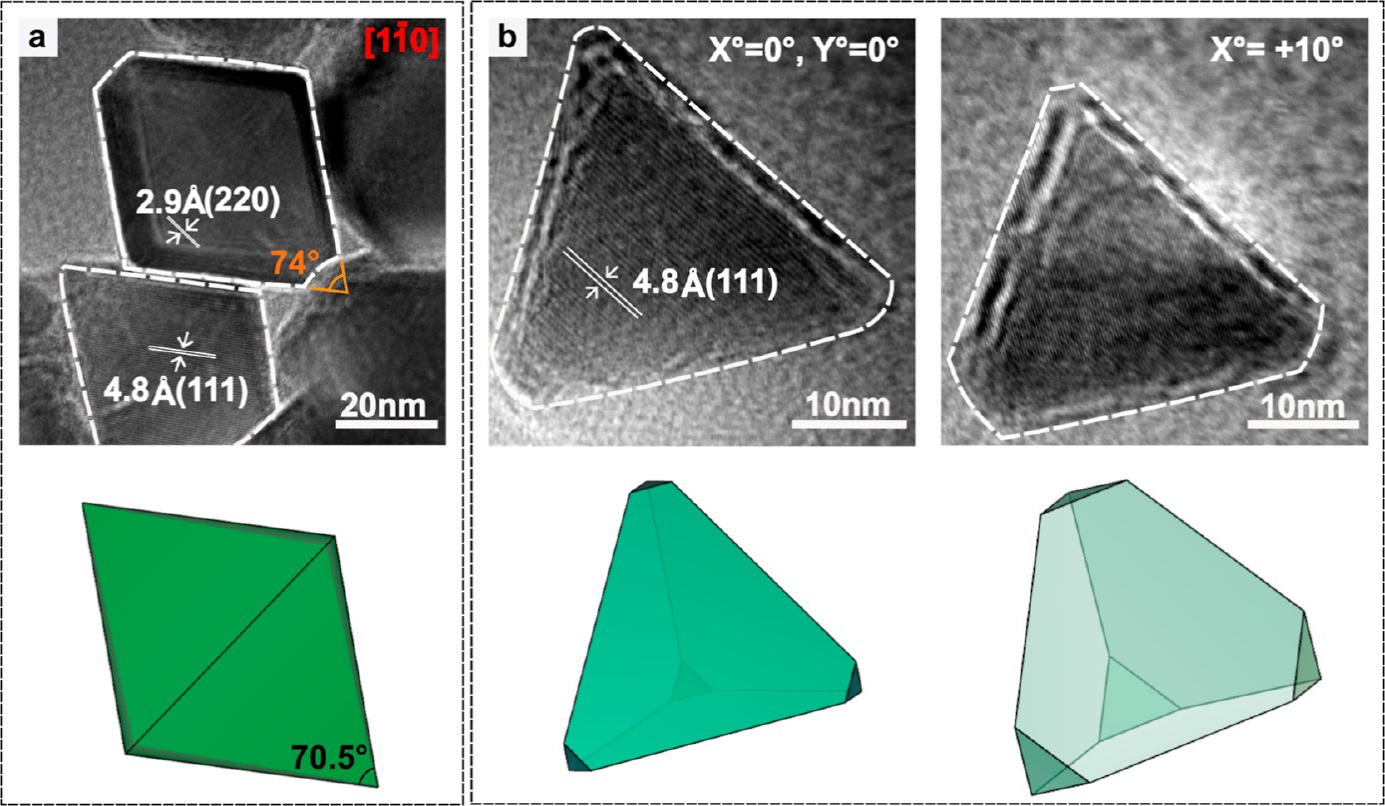
HRTEM analysis of CFZ MNPs synthesized at a reduced amount of OA in the absence and presence of NaOL. (a) HRTEM image of particles synthesized at 2.5 mmol OA, viewed along the [11̅0] zone axis (shown in red), where the octahedral morphology appears as a rhombus. The visible lattice spacing corresponds to (220) and (111) planes, with an interfacial angle of 74° between two (111) facets, estimated by extending the projected edges of the octahedral faces. (b) HRTEM images of particles synthesized at 1.0 mmol NaOL +2.5 mmol OA, shown for various tilt angles in respect to *X* and *Y* axes. The 3D schematics show the orientation of the particular facets contributing to the 2D projections obtained by HRTEM.

### Similar Zn and Co Doping Levels Lead to Highest Magnetization and Lowest Coercivity

To understand how differently formed precursors influence the doping level of Co and Zn in MNPs and how this reflects in their magnetic properties, we performed inductively coupled plasma optical emission spectroscopy (ICP-OES) and field-dependent magnetic measurements using a Magnetic Property Measurement System (MPMS, Quantum Design) at 298 (Figure 9a) and 5 K (Figure 9b). The composition of mixed metal-ferrites and the key parameters of the hysteresis loops (maximum magnetization, *M*_max_, coercive field, *H*_c_) are summarized in Table 1. Although the nominal feed ratios of all three metal ions remain constant across all samples, the actual Fe, Co, and Zn doping levels in the particle chemical formula Co_*x*_Zn_*y*_Fe_3‑(*x*+*y*)_O_4_ vary. To reflect accurately the stoichiometric ratios of the doped metal ions within the spinel lattice, the mass-based concentrations obtained from ICP-OES measurements were converted to atomic percent (atom %) for each cation (Figure 9c–f). The atomic % of both Co and Zn shows a similar trend as a function of the NaOL/OA ratio. The Co atomic content increases first up to 10.5% at the NaOL/OA ratio of 0.2 and then decreases as the ratio decreases. The Zn atomic content increases first to 12.5% at the ratio of 0.4, and then follows a trend similar to that observed for Co. These peculiar doping variations are manifested in the magnetic characteristics of the particles. Looking at the magnetization hysteresis loops recorded at 298 and 5 K, we observed that *M*_max_ increases initially along with Co and Zn content before gradually decreasing as Co and Zn content decrease (Figure 9c,d). As the Co and Zn atomic % increases from CFZ1 (Co_0.3_ Fe_2.5_ Zn_0.2_ O_4_) to CFZ2 (Co_0.33_ Fe_2.29_ Zn_0.38_ O_4_), *M*_max_ increases from 56.5 ± 1.6 emu/g (∼84 emu/g *M*_max_ per Fe + Co) to 72.4 ± 0.9 emu/g (∼116 emu/g *M*_max_ per Fe + Co) at 298 K. The CFZ3 (Co_0.27_ Fe_2.3_ Zn_0.43_ O_4_) sample, which contains the highest zinc (∼12.5 atom %) and relatively low Co content (∼8.7 atom %), as indicated from the ICP data, exhibits a maximum magnetization of 86.03 ± 1.03 emu/g (∼141 emu/g *M*_max_ per Fe + Co) at 298 K and 117.4 ± 3 emu/g at 5 K. These results agree well with previous studies on magnetite^67^ and cobalt-ferrite^68–70^ NPs, which also reported increased *M*_max_ with Zn^2+^ substitution due to cation rearrangement in the spinel lattice. Since Zn^2+^ preferentially occupies tetrahedral (*T*_d_) sites, it weakens the antiferromagnetic coupling between Fe^3+^ in the tetrahedral and octahedral (O_h_) sites and increases the *M*_max_ value.^50,70–72^

**Figure 9:**
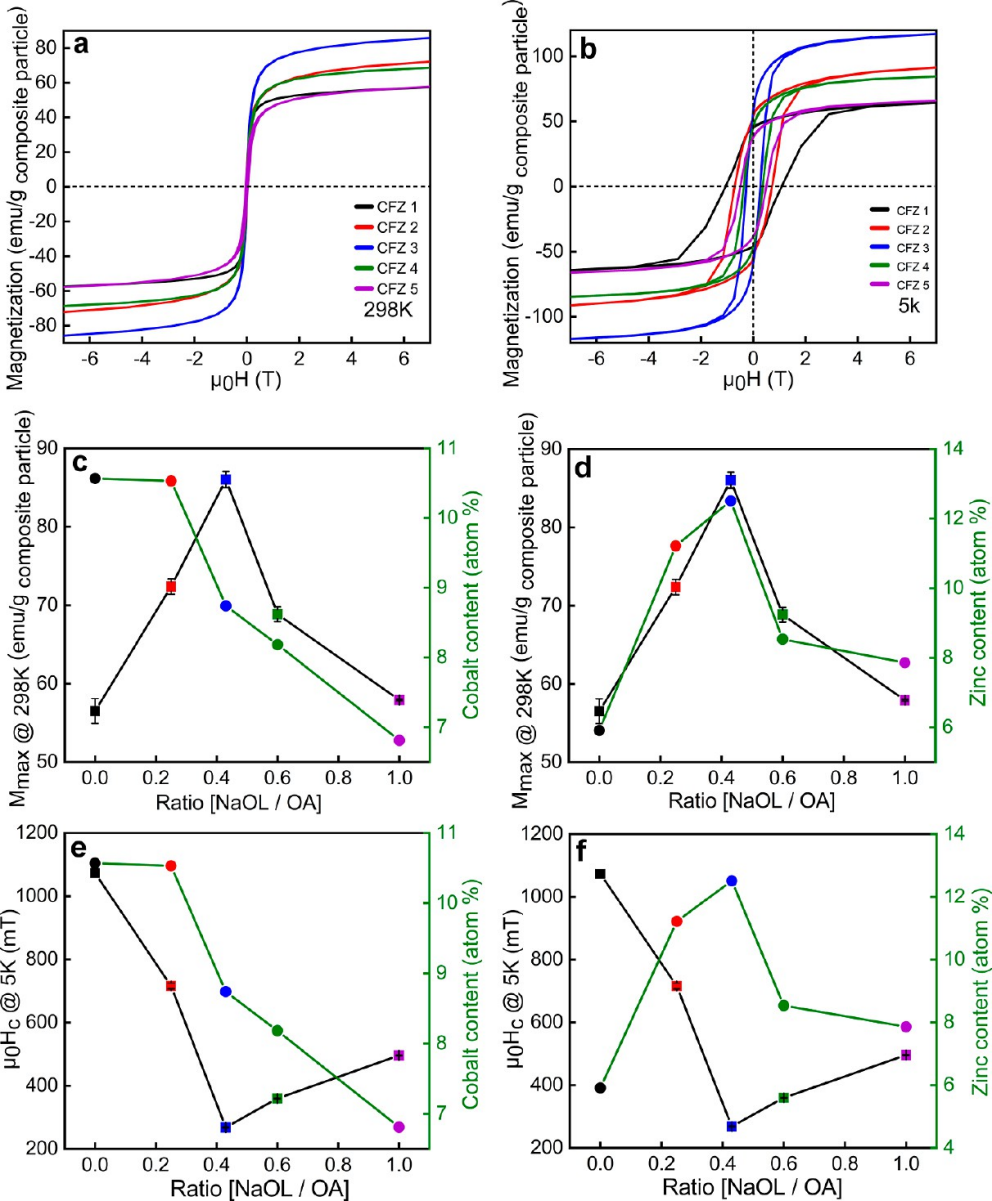
Field-dependent magnetization measurements and analyses. Magnetization hysteresis loops measured at 298 (a) and 5 K (b) and plotted as an average of three independent measurements. The relative standard deviation for all samples ranged from 0.19% to 2.83%. Maximum magnetization (*M*_max_) at 298 K as a function of cobalt content (c) and zinc content (d) with respect to the ligand’s ratio. Coercive field (Hc) at 5 K as a function of cobalt content (e) and zinc content (f) with respect to the ligand’s ratio. The values are mean ± SD and were determined from three independent measurements.

Further increase in the NaOL/OA ratio results in reduced Co and Zn atomic contents in CFZ4 (Co_0.25_ Fe_2.45_ Zn_0.3_ O_4)_ and CFZ5 (Co_0.21_ Fe_2.52_ Zn_0.27_ O_4_); *M*_max_ also drops with values of 68.8 ± 0.9 emu/g (∼107 emu/g *M*_max_ Fe + Co) and 57.9 ± 0.1 emu/g, respectively. This trend exhibits a strong correlation between *M*_max_, Zn, and Co doping level, with the optimal Zn atomic % of 12.5 enhancing the magnetization, while deviations from this composition leads to reduced *M*_max_. These observations fit to our above discussion regarding the distribution of Zn^2+^ and Co^2+^ in the iron oxide crystal structure. By reduction of the doping level of Zn^2+^ in *T*_d_ sites, the antiferromagnetic coupling between Fe^3+^ spins in *T*_d_ and O_h_ sites increases, thus reducing the overall magnetization. Similarly, the reduction of Co^2+^ in the structure by increasing the NaOL/OA ratio from 0.4 to 1 reduces the magnetization, as the particles contain fewer magnetic cations. Revisiting the inverse FFT analysis of nanorods in CFZ5, we note the presence of microstrain (CFZ5, Figure 2), which can contribute to lattice distortions and imperfections that impede the alignment of magnetic moment. This results in a reduction of *M*_max_, in agreement with the findings of Hadadian et al.^73^ Despite the above discussions, one important parameter that plays a crucial role in determining *M*_max_ is the particle size. It is well established that *M*_max_ generally increases with the particle size and approaches saturation when the particle size >100 nm,^74^ yet with some exceptions in Fe_3_O_4_ NPs.^75^ Complete decoupling of effects of size from composition (doping) is indeed challenging in our system, as our current synthesis does not allow the change of the morphology while maintaining the same particle size. Considering our previous discussion on the composition of doped ferrites and including the effect of particle size, the following observations can be made. A synergistic effect between *D*_eff_^norm.^ size (Figure S1) and Zn^2+^/Co^2+^ doping levels was observed with increasing NaOL/OA ratio up to 0.4, as both parameters increase simultaneously. The larger particle size thus may have a positive contribution to the increased *M*_max_ values in this range. However, when the NaOL/OA ratio further increases from 0.4 to 1 (CFZ4 & CFZ5), particle size continues to grow while the Zn^2+^ and Co^2+^ doping level decrease. Although an increased amount of NaOL promotes more zinc oleate formation, there is a threshold beyond which zinc incorporation into the particle crystal structure begins to decrease, as indicated by the ICP and magnetic measurements.

The ferri­(o)­magnetic character of the samples can be gathered by the presence of coercivity (μ_0_ *H*_c_) at RT for all samples. The μ_0_ *H*_c_ observed at both 298 and 5 K reflects the complex interplay between particle morphology, magnetic anisotropy, and cation distribution. The effect of the Co doping level on μ_0_ *H*_c_, as shown in Figure 9e, exhibits complex behavior. Although Co^2+^ generally enhances anisotropy and increases coercivity, our samples first show a decrease in μ_0_ *H*_c_ with the Co content up to a NaOL/OA ratio of 0.2, followed by an increase in μ_0_ *H*_c_ as the Co content gradually decreases. μ_0_ *H*_c_ reaches the lowest value in CFZ3 up to a NaOL/OA ratio of 0.4 (Figure 9e), primarily due to the reduced Co^2+^, which reduces the magnetocrystalline anisotropy and leads to magnetic softening. Beyond this ratio, μ_0_H_c_ rises, suggesting the dominance of Co in the anisotropy contribution as the Zn doping level drops.

The formation mechanisms of kinetic morphologies in noble metal NPs are well-understood, with established growth models highlighting the role of complex precursors on reaction kinetics^40^ and ligand-guided facet stabilization.^49^ In contrast, the current understanding of how kinetically controlled morphologies in metal oxides can be synthesized remains limited, despite morphologies such as nanodiscs being very appealing for magneto-mechanical manipulation of cellular processes and the destruction of cancer cells.^76^ Tetrahedral MNPs with significantly larger surface area-to-volume ratio and reduced (*T*_d_) symmetry compared to those of octahedrons (O_h_) cannot be formed in thermodynamically controlled synthesis.^36,40^ Our results demonstrate that by tuning the ligand chemistry, we can navigate the fine boundary between thermodynamically and kinetically controlled growth pathways in tricomponent MNPs. Specifically, when OA is used as a ligand, we observe the formation of thermodynamically stable octahedra enclosed entirely by low-energy {111} facets, as monomer deposition rates are driven by surface energy minimization, favoring symmetric shapes. The synthesis of octahedra does not further continue down the typical pathway by growing and vanishing (111) planes and forming cubic MNPs. This is presumably due to two concurring processes. First, it is known that the monomer concentration drops rapidly in thermal decomposition syntheses. Therefore, it may well be that after an initial rapid growth step, there is not enough monomer left. The second mechanism is the strong passivation of {111} facets by OA, thus halting the monomers from further accumulating on these facets.

The addition of NaOL to the synthesis shifts the growth regime toward kinetic control, enabling the formation of tetrahedrons. Our extensive HRTEM investigations unambiguously show that the tetrahedrons are enclosed by four (111) planes. Increasing NaOL concentrations leads to stochastic and extreme truncation of {111} planes and the formation of extremely truncated tetrahedra, all the way up to rod-like shapes. We show that the synthesis of Co- and Zn-doped iron oxide MNPs with tetrahedral morphology does not require a symmetry-breaking transformation from octahedra, as previously proposed by Xia et al.^37^ but instead can be directed by tuning ligand composition to include NaOL. Kinetic morphologies of MNPs have thus far only been shown for iron oxide NPs. Kovalenkov et al. synthesized bipyramidal iron oxide nanocrystals by tuning mmol ratios of NaOL to FeOL,^32^ whereas Zhou et al. demonstrated the formation of various IONPs shapes, including plates, truncated octahedra, and tetrahedra, by varying the ratios of NaOL to FeOL and keeping the amount of OA constant.^28^ Additionally, Qiao et al. reported the synthesis of tetrahedral and octahedral IONPs by introducing benzaldehyde into the system,^62^ emphasizing the importance of ligand-mediated control over nanoparticle morphology. While we achieved rod-shaped morphology in ternary MNPs by varying ligands ratio, Khurshid et al. obtained iron oxide nanorods by applying a slower heating rate.^77^ Our study offers novel mechanistic insights into how to synthesize such morphologies in ternary MNPs with high saturation magnetization and tunable magnetic coercivity.

## Conclusion

Here, we present mechanistic insights into the growth mechanism of kinetic morphologies of Co- and Zn-doped iron oxide MNPs by varying the molar ratios of two commonly used ligands, OA and NaOL. Our findings reveal that adjusting ligand ratios significantly alters the precursor chemistry, with the effects becoming more pronounced at reduced OA and increased NaOL concentrations. These changes influence the nucleation dynamics and shift the growth mechanism from thermodynamically to kinetically driven pathways. Compared to the complex two-step seed-and-growth approaches that are often employed in the synthesis of tetrahedron-shaped noble metal NPs^39,40^ and to generate complex morphologies in doped iron oxide MNPs,^64^ our study underscores a simple strategy to form tetrahedra and extremely truncated tetrahedra resembling rod-shaped MNPs in complex composition of mixed metal ferrite MNPs by including NaOL into the synthesis. Our study highlights NaOL’s role as a capping ligand for the facet-selective and extreme passivation of {111} crystal facets resulting in the formation of tetrahedrons and rod-shaped MNPs. Furthermore, the precursor composition also affects the doping efficiency of Co and Zn ions into the ferrite crystal structure. Our FTIR results indicate that NaOL promotes the formation of zinc oleate. Although an increase in the NaOL concentration from CFZ2 to CFZ5 might suggest an increase in the Zn doping level across the series, instead, a reduction in the zinc doping level is observed. This is attributed to the reduced OA concentration, as it is being compensated with NaOL’s increased involvement in the formation of bimetallic complexes along with zinc oleate as the precursor. With a maximum zinc doping (atomic % of 12.5) in the tetrahedral sites of inverse spinel lattice, the tetrahedron-shaped MNPs (CFZ3) exhibit the highest magnetization among other samples at RT, contributing to *M* of 86 emu/g of composite particle, equivalent to 141 emu/g of magnetic material (Fe + Co) recorded at 7 T. The *M*_max_ value obtained for CFZ3 agrees well with previously reported magnetization values for Co–Zn ferrites with similar compositions.^70,78^ This strong magnetic performance makes them a promising candidate for biomedical applications. Owing to their large surface area, tetrahedron-shaped MNPs offer a distinct advantage over cubic and spherical counterparts, as they can have a higher loading of functional polymers and biomarkers, thereby reducing the required MNP concentration in bioassays without compromising sensitivity. Upon transfer to an aqueous medium as a single, colloidally stable particle, these MNPs can be effectively used in magnetic bioassays, where Brownian relaxation behavior of MNPs is critical for detecting analyte binding events.

## Supplementary Material



## Notes

Schematic illustrations of the procedure used to estimate the effective nominal particle size (*D*_eff_^nom.^) from TEM images, size and morphology distribution histograms, 2D projections of synthesized nanoparticles along different crystallographic directions, TGA and DTG curves of zinc oleate precursors, and HAADF-STEM images of kinetic morphologies obtained as a result of varying NaOL to OA ratios (PDF)

## References

[1] Tian B., Ma J., Qiu Z., Zardán Gómez de la Torre T., Donolato M., Hansen M. F., Svedlindh P., Strömberg M. (2017). Optomagnetic detection of microRNA based on duplex-specific
nuclease-assisted target recycling and multilayer core-satellite magnetic
superstructures. ACS Nano.

[2] Wu K., Su D., Saha R., Liu J., Wang J. P. (2019). Investigating
the
effect of magnetic dipole–dipole interaction on magnetic particle
spectroscopy: implications for magnetic nanoparticle-based bioassays
and magnetic particle imaging. J. Phys. D: Appl.
Phys..

[3] Rösch E. L., Sack R., Chowdhury M. S., Wolgast F., Zaborski M., Ludwig F., Schilling M., Viereck T., Rand U., Lak A. (2024). Amplification-and Enzyme-Free
Magnetic Diagnostics Circuit for Whole-Genome
Detection of SARS-CoV-2 RNA. ChemBioChem.

[4] Cho M. H., Lee E. J., Son M., Lee J. H., Yoo D., Kim J. W., Park S. W., Shin J. S., Cheon J. (2012). A magnetic
switch for the control of cell death signalling in in vitro and in
vivo systems. Nat. Mater..

[5] Chen R., Romero G., Christiansen M. G., Mohr A., Anikeeva P. (2015). Wireless magnetothermal
deep brain stimulation. Science.

[6] Krishnan K. M. (2010). Biomedical
nanomagnetics: a spin through possibilities in imaging, diagnostics,
and therapy. IEEE Trans. Magn..

[7] Muro-Cruces J., Roca A. G., López-Ortega A., Fantechi E., del-Pozo-Bueno D., Estradé S., Peiró F., Sepúlveda B., Pineider F., Sangregorio C. (2019). Precise size control
of the growth of Fe3O4 nanocubes over a wide size range using a rationally
designed one-pot synthesis. ACS Nano.

[8] Sharifi
Dehsari H., Halda Ribeiro A., Ersöz B., Tremel W., Jakob G., Asadi K. (2017). Effect of precursor
concentration on size evolution of iron oxide nanoparticles. CrystEngComm.

[9] Hyeon T., Chung Y., Park J., Lee S. S., Kim Y. W., Park B. H. (2002). Synthesis of highly crystalline and monodisperse cobalt
ferrite nanocrystals. J. Phys. Chem. B.

[10] Johnson M. K., Powell D. B., Cannon R. D. (1981). Vibrational
spectra of carboxylato
complexes—III. Trinuclear ‘basic’acetates and
formates of chromium (III), iron (III) and other transition metals. Spectrochim. Acta A Mol. Spectrosc..

[11] Plummer L. K., Hutchison J. E. (2020). Understanding
the effects of iron precursor ligation
and oxidation state leads to improved synthetic control for spinel
iron oxide nanocrystals. Inorg.Chem..

[12] Kemp S. J., Ferguson R. M., Khandhar A. P., Krishnan K. M. (2016). Monodisperse magnetite
nanoparticles with nearly ideal saturation magnetization. RSC Adv..

[13] Palii S. P., Richardson D. E., Hansen M. L., Iversen B. B., Larsen F. K., Sîngerean L., Timco G. A., Gerbeleu N. V., Jennings K. R., Eyler J. R. (2001). Mixed-terminal-ligand oxo-centered
carboxylate-bridged trinuclear complexes: gas phase generation by
means of electrospray ionization FT-ICR MS, condensed phase synthesis,
and X-ray structure of K+ [Cr3O (C6H5COO) 6 (F) 2 (H2O)]–·
2 (CH3) 2CO. Inorg. Chim. Acta.

[14] Rowell J. L., Jia Y., Shi Z., Molina Villarino A., Kang M., Yoon D., Jiang K. Z., Abruña H. D., Muller D. A., Robinson R. D. (2023). General
route to colloidally stable, low-dispersity manganese-based ternary
spinel oxide nanocrystals. J. Am. Chem. Soc..

[15] Rowell J. L., Kang M., Yoon D., Jiang K. Z., Jia Y., Abruña H. D., Muller D. A., Robinson R. D. (2024). Colloidal synthesis
of monodisperse high-entropy spinel oxide nanocrystals. J. Am. Chem. Soc..

[16] Chang H., Kim B. H., Lim S. G., Baek H., Park J., Hyeon T. (2021). Role of the precursor composition in the synthesis of metal ferrite
nanoparticles. Inorg. Chem..

[17] Bao N., Shen L., Wang Y., Padhan P., Gupta A. (2007). A facile thermolysis
route to monodisperse ferrite nanocrystals. J. Am. Chem. Soc..

[18] Boyle T. J., Bunge S. D., Andrews N. L., Matzen L. E., Sieg K., Rodriguez M. A., Headley T. J. (2004). Precursor Structural Influences on
the Final ZnO Nanoparticle Morphology from a Novel Family of Structurally
Characterized Zinc Alkoxy Alkyl Precursors. Chem. Mater..

[19] Puzder A., Williamson A. J., Zaitseva N., Galli G., Manna L., Alivisatos A. P. (2004). The effect
of organic ligand binding on the growth
of CdSe nanoparticles probed by ab initio calculations. Nano Lett..

[20] Pearson R. G. (1963). Hard and
soft acids and bases. J. Am. Chem. Soc..

[21] Kwon S. G., Piao Y., Park J., Angappane S., Jo Y., Hwang N. M., Park J. G., Hyeon T. (2007). Kinetics of monodisperse
iron oxide nanocrystal formation by “heating-up” process. J. Am. Chem. Soc..

[22] Niederberger M. (2007). Nonaqueous
sol–gel routes to metal oxide nanoparticles. Acc. Chem. Res..

[23] Kwon S. G., Hyeon T. (2008). Colloidal chemical synthesis and formation kinetics of uniformly
sized nanocrystals of metals, oxides, and chalcogenides. Acc. Chem. Res..

[24] An K., Somorjai G. A. (2012). Size and shape control of metal nanoparticles for reaction
selectivity in catalysis. ChemCatChem.

[25] Yang T. H., Shi Y., Janssen A., Xia Y. (2020). Surface capping agents and their
roles in shape-controlled synthesis of colloidal metal nanocrystals. Angew. Chem., Int. Ed..

[26] Wu L., Mendoza-Garcia A., Li Q., Sun S. (2016). Organic phase syntheses
of magnetic nanoparticles and their applications. Chem. Rev..

[27] Ding X., Bao L., Jiang J., Gu H. (2014). Colloidal synthesis of ultrathin
γ-Fe 2 O 3 nanoplates. RSC Adv..

[28] Zhou Z., Zhu X., Wu D., Chen Q., Huang D., Sun C., Xin J., Ni K., Gao J. (2015). Anisotropic shaped iron oxide nanostructures:
controlled synthesis and proton relaxation shortening effects. Chem. Mater..

[29] Xie J., Yan C., Zhang Y., Gu N. (2013). Shape evolution of
“multibranched”
Mn–Zn ferrite nanostructures with high performance: a transformation
of nanocrystals into nanoclusters. Chem. Mater..

[30] Nader K., Castellanos-Rubio I., Orue I., Iglesias-Rojas D., Barón A., Gil de Muro I., Lezama L., Insausti M. (2022). Getting insight
into how iron (III) oleate precursors affect the features of magnetite
nanoparticles. J. Solid State Chem..

[31] Situ-Loewenstein S. F., Wickramasinghe S., Abenojar E. C., Erokwu B. O., Flask C. A., Lee Z., Samia A. C. S. (2018). A novel synthetic
route for high-index faceted iron
oxide concave nanocubes with high T2 relaxivity for in vivo MRI applications. J.Mater.Sci: Mater. Med..

[32] Kovalenko M. V., Bodnarchuk M. I., Lechner R. T., Hesser G., Schäffler F., Heiss W. (2007). Fatty acid salts as stabilizers in size-and shape-controlled nanocrystal
synthesis: the case of inverse spinel iron oxide. J. Am. Chem. Soc..

[33] Wetterskog E., Agthe M., Mayence A., Grins J., Wang D., Rana S., Ahniyaz A., Salazar-Alvarez G., Bergström L. (2014). Precise control over shape and size of iron oxide nanocrystals
suitable for assembly into ordered particle arrays. Sci. Technol. Adv. Mater..

[34] Zhou Z., Zhao Z., Zhang H., Wang Z., Chen X., Wang R., Chen Z., Gao J. (2014). Interplay
between longitudinal
and transverse contrasts in Fe3O4 nanoplates with (111) exposed surfaces. ACS Nano.

[35] Palchoudhury S., Xu Y., Rushdi A., Holler R. A., Bao Y. (2012). Controlled synthesis
of iron oxide nanoplates and nanoflowers. Chem.
Commun..

[36] Sun M., Cheng Z., Chen W., Jones M. (2021). Understanding symmetry
breaking at the single-particle level via the growth of tetrahedron-shaped
nanocrystals from higher-symmetry precursors. ACS Nano.

[37] Xia Y., Nelli D., Ferrando R., Yuan J., Li Z. Y. (2021). Shape control
of size-selected naked platinum nanocrystals. Nat. Commun..

[38] Kim F., Connor S., Song H., Kuykendall T., Yang P. (2004). Platonic gold nanocrystals. Angew. Chem., Int.
Ed..

[39] Zheng Y., Liu W., Lv T., Luo M., Hu H., Lu P., Choi S., Zhang C., Tao J., Zhu Y. (2014). Seed-mediated synthesis of gold tetrahedra
in high purity and with
tunable, well-controlled sizes. Chem.—Asian
J..

[40] Wang Y., Xie S., Liu J., Park J., Huang C. Z., Xia Y. (2013). Shape-controlled
synthesis of palladium nanocrystals: a mechanistic understanding of
the evolution from octahedrons to tetrahedrons. Nano Lett..

[41] Biacchi A.
J., Schaak R. E. (2011). The solvent
matters: kinetic versus thermodynamic shape
control in the polyol synthesis of rhodium nanoparticles. ACS Nano.

[42] Lalegani Z., Ebrahimi S. S. (2020). Optimization of synthesis for shape and size controlled
silver nanoparticles using response surface methodology. Colloids Surf. A Physicochem. Eng. Asp..

[43] Zong R., Wang X., Shi S., Zhu Y. (2014). Kinetically controlled
seed-mediated growth of narrow dispersed silver nanoparticles up to
120 nm: secondary nucleation, size focusing, and Ostwald ripening. Phys. Chem. Chem. Phys..

[44] Chowdhury M. S., Rösch E. L., Esteban D. A., Janssen K. J., Wolgast F., Ludwig F., Lak A. (2022). Decoupling the characteristics of
magnetic nanoparticles for ultrahigh sensitivity. Nano Lett..

[45] Zhou H., Lv B., Wu D., Xu Y. (2013). Synthesis and properties of octahedral
Co 3 O 4 single-crystalline nanoparticles enclosed by (111) facets. CrystEngComm.

[46] Castellanos-Rubio I., Rodrigo I., Munshi R., Arriortua O., Garitaonandia J. S., Martinez-Amesti A., Plazaola F., Orue I., Pralle A., Insausti M. (2019). Outstanding heat loss via nano-octahedra
above 20 nm in size: From wustite-rich nanoparticles to magnetite
single-crystals. Nanoscale.

[47] Wetterskog E., Tai C. W., Grins J., Bergstrom L., Salazar-Alvarez G. (2013). Anomalous magnetic properties of nanoparticles arising
from defect structures: topotaxial oxidation of Fe1–x O| Fe3–
δO4 core| shell nanocubes to single-phase particles. ACS Nano.

[48] Li Z. (2011). Characterization
of Different Shaped Nanocrystallites using X-ray Diffraction Line
Profiles. Part. Part. Syst. Charact..

[49] Mozaffari S., Li W., Thompson C., Ivanov S., Seifert S., Lee B., Kovarik L., Karim A. M. (2017). Colloidal nanoparticle size control:
experimental and kinetic modeling investigation of the ligand–metal
binding role in controlling the nucleation and growth kinetics. Nanoscale.

[50] Tartaj P., Morales M. P., Gonzalez-Carreno T., Veintemillas-Verdaguer S., Serna C. J. (2005). Advances in magnetic nanoparticles for biotechnology
applications. J. Magn. Magn. Mater..

[51] Lak A., Kahmann T., Schaper S. J., Obel J., Ludwig F., Müller-Buschbaum P., Lipfert J. (2020). The dissociation rate
of acetylacetonate ligands governs the size of ferrimagnetic zinc
ferrite nanocubes. ACS Appl. Mater. Interfaces.

[52] Kirkpatrick K. M., Zhou B. H., Bunting P. C., Rinehart J. D. (2022). Size-tunable magnetite
nanoparticles from well-defined iron oleate precursors. Chem. Mater..

[53] Song L., Huo H., Zhang W., Xia H., Niu Y. (2022). The facile strategy
of improving the long-term stability of highly transparent polyvinyl
chloride by introducing unsaturated Zn oleate and uracil derivatives. Materials.

[54] Sepúlveda F. A., Rivera F., Loyo C., Canales D., Moreno-Serna V., Benavente R., Rivas L. M., Ulloa M. T., Gil-Castell O., Ribes-Greus A. (2022). Poly (lactic acid)/D-limonene/ZnO bio-nanocomposites
with antimicrobial properties. J. Appl. Polym.
Sci..

[55] Om, F. ; Ayinde, A. A. Thermal Stability Study of s (Ricinuscommunis. Res. J. Chem. Sci. 2016.

[56] Morrow L., Barron A. R. (2015). Issues affecting the synthetic scalability of ternary
metal ferrite nanoparticles. J. Nanoparticle..

[57] Castellanos-Rubio I., Arriortua O., Iglesias-Rojas D., Barón A., Rodrigo I., Marcano L., Garitaonandia J. S., Orue I., Fdez-Gubieda M. L., Insausti M. (2021). A milestone in the
chemical synthesis of Fe3O4 nanoparticles: unreported bulklike properties
lead to a remarkable magnetic hyperthermia. Chem. Mater..

[58] Ozel F., Kockar H., Beyaz S., Karaagac O., Tanrisever T. (2013). Superparamagnetic
iron oxide nanoparticles: effect of iron oleate precursors obtained
with a simple way. J. Mater. Sci.: Mater. Electron..

[59] Bronstein L. M., Huang X., Retrum J., Schmucker A., Pink M., Stein B. D., Dragnea B. (2007). Influence
of iron oleate
complex structure on iron oxide nanoparticle formation. Chem. Mater..

[60] Feld A., Weimer A., Kornowski A., Winckelmans N., Merkl J. P., Kloust H., Zierold R., Schmidtke C., Schotten T., Riedner M. (2018). Chemistry of shape-controlled
iron oxide nanocrystal formation. ACS Nano.

[61] Thanh N. T., Maclean N., Mahiddine S. (2014). Mechanisms
of nucleation and growth
of nanoparticles in solution. Chem. Rev..

[62] Qiao L., Fu Z., Li J., Ghosen J., Zeng M., Stebbins J., Prasad P. N., Swihart M. T. (2017). Standardizing size-and shape-controlled
synthesis of monodisperse magnetite (Fe3O4) nanocrystals by identifying
and exploiting effects of organic impurities. ACS Nano.

[63] Narnaware P. K., Ravikumar C. (2020). Mechanistic Insights into the Formation and Growth
of Anisotropic-Shaped Wüstite–Spinel Core–Shell
Iron Oxide Nanoparticles in a Coordinating Solvent. J. Phys. Chem. C.

[64] Singh P., Duraisamy K., Raitmayr C., Sharma K. S., Korzun T., Singh K., Moses A. S., Yamada K., Grigoriev V., Demessie A. A. (2025). Precision-Engineered
Cobalt-Doped Iron Oxide
Nanoparticles: From Octahedron Seeds to Cubical Bipyramids for Enhanced
Magnetic Hyperthermia. Adv. Funct. Mater..

[65] Lee J. U., Shin W., Lim Y., Kim J., Kim W. R., Kim H., Lee J. H., Cheon J. (2021). Non-contact long-range magnetic stimulation
of mechanosensitive ion channels in freely moving animals. Nat. Mater..

[66] López-Ortega A., Lottini E., Fernandez C. D. J., Sangregorio C. (2015). Exploring
the magnetic properties of cobalt-ferrite nanoparticles for the development
of a rare-earth-free permanent magnet. Chem.
Mater..

[67] Bohara R. A., Thorat N. D., Chaurasia A. K., Pawar S. H. (2015). Cancer cell extinction
through a magnetic fluid hyperthermia treatment produced by superparamagnetic
Co–Zn ferrite nanoparticles. RSC Adv..

[68] Jang J. T., Nah H., Lee J. H., Moon S. H., Kim M. G., Cheon J. (2009). Critical enhancements
of MRI contrast and hyperthermic effects by dopant-controlled magnetic
nanoparticles. Angew. Chem., Int. Ed..

[69] Lin Q., Xu J., Yang F., Lin J., Yang H., He Y. (2018). Magnetic and
Mössbauer spectroscopy studies of zinc-substituted cobalt ferrites
prepared by the sol-gel method. Materials.

[70] Baričić M., Maltoni P., Barucca G., Yaacoub N., Omelyanchik A., Canepa F., Mathieu R., Peddis D. (2024). Chemical engineering
of cationic distribution in spinel ferrite nanoparticles: the effect
on the magnetic properties. Phys. Chem. Chem.
Phys..

[71] Iglesias-Rojas D., Nader K., Fernández-Lavilla N., Mentxaka-Salgado J., Gil de Muro I., Garitaonandia J. S., Orue I., Castellanos-Rubio A., Insausti M., Castellanos-Rubio I. (2025). From Bimetallic Oleates to Customized
Biomedical Nanoplatforms: A Versatile Approach for the Multidoping
of Ferrites. ACS Appl. Mater. Interfaces.

[72] Albino M., Fantechi E., Innocenti C., López-Ortega A., Bonanni V., Campo G., Pineider F., Gurioli M., Arosio P., Orlando T. (2019). Role of
Zn2+ substitution
on the magnetic, hyperthermic, and relaxometric properties of cobalt
ferrite nanoparticles. J. Phys. Chem. C.

[73] Hadadian Y., Masoomi H., Dinari A., Ryu C., Hwang S., Kim S., Cho B. k., Lee J. Y., Yoon J. (2022). From low to high saturation
magnetization in magnetite nanoparticles: the crucial role of the
molar ratios between the chemicals. ACS omega.

[74] Li Q., Kartikowati C. W., Horie S., Ogi T., Iwaki T., Okuyama K. (2017). Correlation
between particle size/domain structure
and magnetic properties of highly crystalline Fe3O4 nanoparticles. Sci. Rep..

[75] Chen F., Ilyas N., Liu X., Li Z., Yan S., Fu H. (2021). Size effect of Fe3O4 nanoparticles
on magnetism and dispersion stability
of magnetic nanofluid. Front. Energy. Res..

[76] Gregurec D., Senko A. W., Chuvilin A., Reddy P. D., Sankararaman A., Rosenfeld D., Chiang P. H., Garcia F., Tafel I., Varnavides G. (2020). Magnetic vortex nanodiscs
enable remote magnetomechanical
neural stimulation. ACS Nano.

[77] Khurshid H., Li W., Chandra S., Phan M. H., Hadjipanayis G. C., Mukherjee P., Srikanth H. (2013). Mechanism and controlled
growth of
shape and size variant core/shell FeO/Fe 3 O 4 nanoparticles. Nanoscale.

[78] Mameli V., Musinu A., Ardu A., Ennas G., Peddis D., Niznansky D., Sangregorio C., Innocenti C., Thanh N. T. K., Cannas C. (2016). Studying the effect of Zn-substitution
on the magnetic and hyperthermic properties of cobalt ferrite nanoparticles. Nanoscale.

