# Supplementary material for: Kinetically Controlled Morphologies of Magnetic Nanoparticles through Ligand and Precursor Chemistry
Source: ACS Nanosci Au. 2025 Nov 5;6(1):68–85. doi: 10.1021/acsnanoscienceau.5c00099 (PMC12921614; doi:10.1021/acsnanoscienceau.5c00099)
Supplement: Supplementary file 1 [file ng5c00099_si_001.pdf]

Supporting Information

Kinetically-Controlled Morphologies of Magnetic Nanoparticles through Ligand and Precursor Chemistry

Rabia Amin[1], Yihao Wang[1], Johannes Berlin[2], Markus Etzkorn[3], Christopher R. Everett[4], Susanne Kempter[5], Meinhard Schilling[1], Peter Müller-Buschbaum[4], Jan Lipfert[2], Mohammad Suman Chowdhury[1], Aidin Lak[1*]

[1]Institute for Electrical Measurement Science and Fundamental Electrical Engineering and Laboratory for Emerging Nanometrology (LENA), Hans-Sommer-Str. 66, Braunschweig, 38106, Germany.

[2]Institute of Physics, University of Augsburg, Universitätsstraße 1, 86159 Augsburg, Germany.

[3]Institute of Applied Physics, TU Braunschweig, Mendelssohnstraße 2, 38106, Braunschweig, Germany.

[4]Technical University of Munich, TUM School of Natural Sciences, Department of Physics, Chair for Functional Materials, James-Franck-Str. 1, 85748 Garching, Germany.

[5]Department of Physics and Center for NanoScience, LMU Munich, Amalienstrasse 54, 80539 Munich, Germany.

Email: a.lak@tu-braunschweig.de

Estimation of $D_{\mathrm{eff}}^{\mathrm{nom.}}$ along the normal of diffraction plane: Defining the physical particle size for different morphologies such as octahedron, truncated tetrahedron with different edge truncations and elongations, is rather challenging. Consequently, a direct comparison between the TEM-measured particle size and the effective crystallite size obtained from XRD ($D_{\mathrm{eff}}^{\mathrm{XRD}}$) is not straightforward. To obtain a consistent and shape-independent size index for such morphologies, we converted the particle size measured along the normal of the (220) diffraction planes from TEM to the effective nominal crystallite size ($D_{\mathrm{eff}}^{\mathrm{nom.}}$). For octahedral particles, the apparent edge length (AEL) measured from TEM corresponds directly to the edge length of a perfectly formed octahedron. To more accurately determine the physical particle size for complex morphologies such as, truncated tetrahedron, the true edge length of the ideal polyhedron (represented as 'a' in Figure S1) was first reconstructed by summing the AEL with the truncation lengths measured along the same direction. This reconstructed edge length was then converted to the edge length L of a cube that is circumscribed to a polyhedron, following the approach of Li[48]. The nominal crystallite size ($D_{\mathrm{eff}}^{\mathrm{nom.}}$) along the [220] crystalline direction was then calculated as $D_{\mathrm{eff}}^{\mathrm{nom.}} = 0.530 \times L$ for octahedron, and $D_{\mathrm{eff}}^{\mathrm{nom.}} = 0.707 \times L$ for tetrahedron.

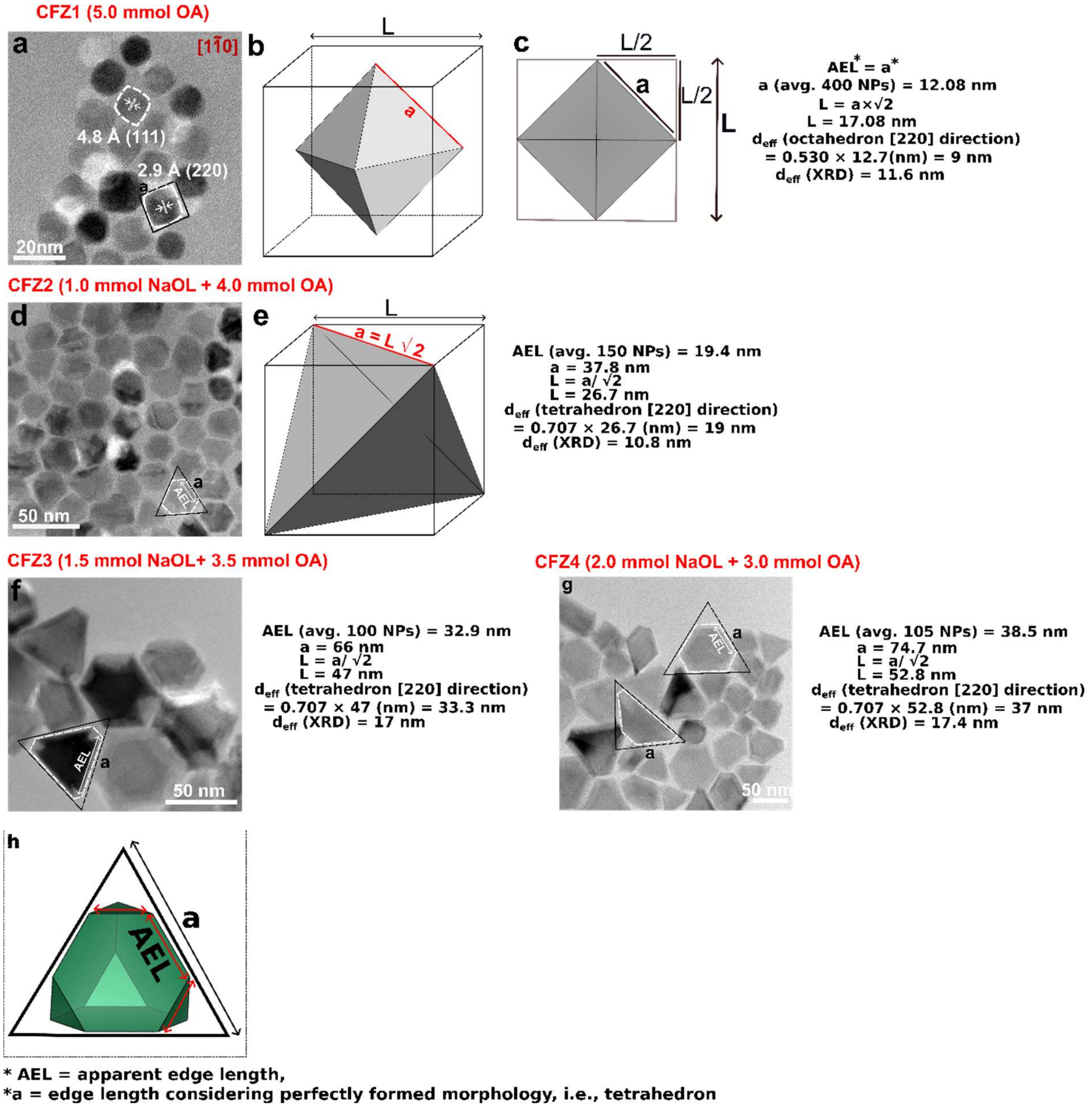

Figure S1. Schematic illustration of the procedure used to estimate the effective nominal crystallite size ($D_{eff}^{norm.}$) along the normal of (220) planes from TEM images. (a) HRTEM image of CFZ1 NPs and contours of two typically oriented octahedra on gird. (b) The circumscribed cube with the edge length of L to an octahedron is shown. (c) The relation between a (which corresponds to edge length of perfectly formed polyhedra) and L. (d) HRTEM image of CFZ2 truncated tetrahedron with its contour considering a perfectly formed tetrahedron. (e) A tetrahedron circumscribed inside a cube with the edge length of L. (f) and (g) HRTEM images of CFZ3 and CFZ4 tetrahedra with their contours considering a perfectly formed tetrahedra, respectively. (h) Schematic showing the apparent edge length and edge length of a perfectly formed tetrahedra.

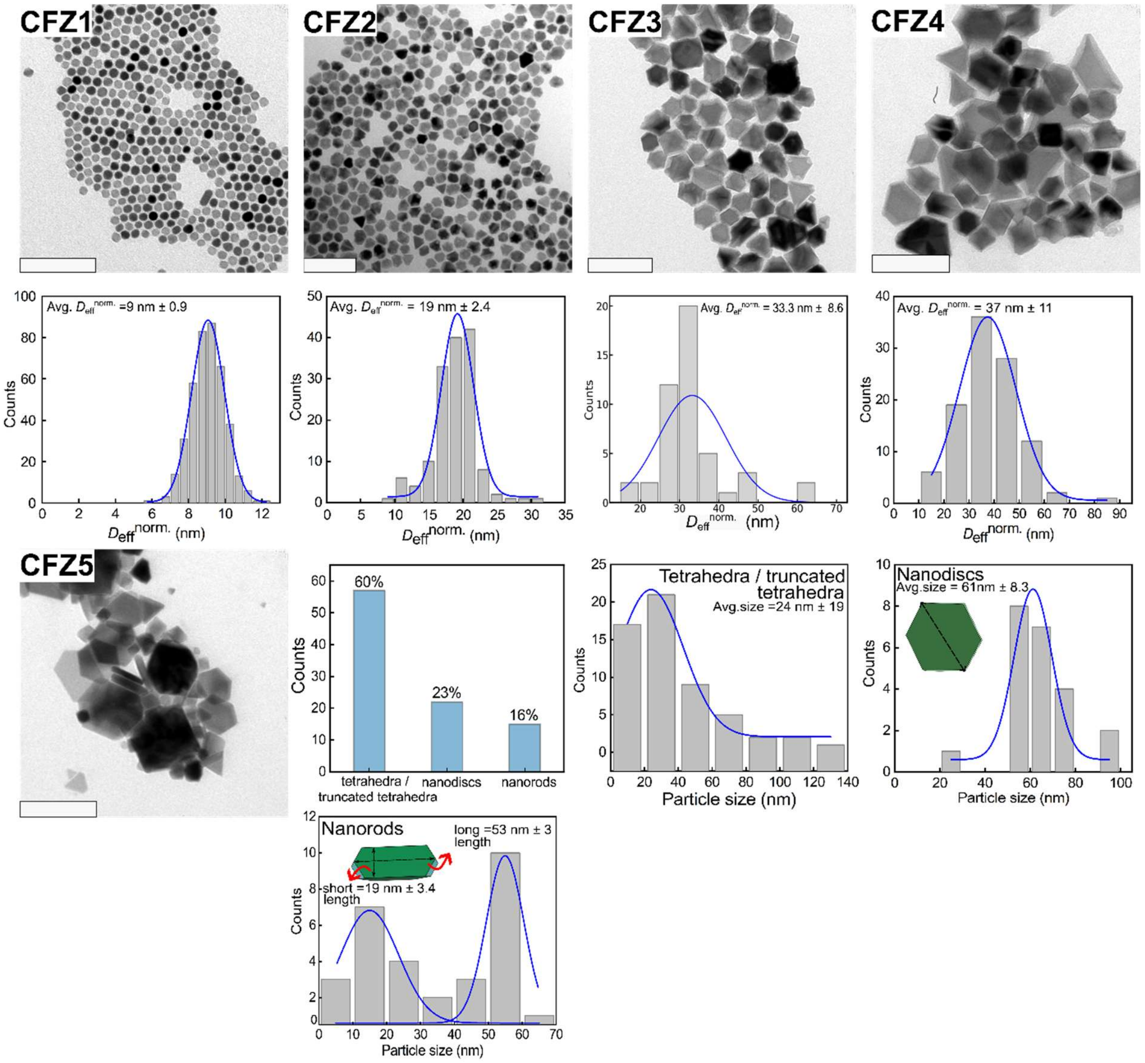

Figure S2. Particle size and morphology distribution analysis. The size analysis was performed by measuring the particle edge length along the [220] direction and converting it into the effective nominal crystallite size ($D_{eff}^{nom.}$), taking particle shape into account. The histograms were generated by counting more than 100 particles. For CFZ5, average particle sizes were determined directly from the apparent edge lengths (AEL) measured from TEM images, and were not converted to $D_{eff}^{nom.}$. The scale bars correspond to 100 nm. The standard deviations are 1σ.

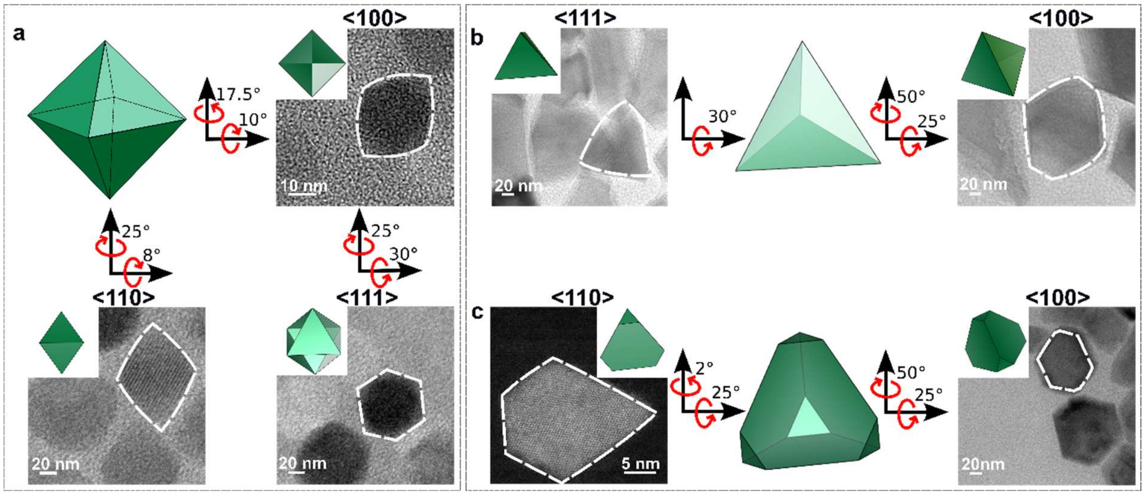

Figure S3. 2D projections of (a) octahedrons, (b) tetrahedrons and (c) truncated tetrahedrons viewed along [100], [110], and [111] crystallographic directions. The corresponding HRTEM images, along with schematic representations illustrate these projections. The detailed rotations are indicated by red arrows along the X and Y axes, with the specified angles showing the applied rotations to achieve the observed orientations.

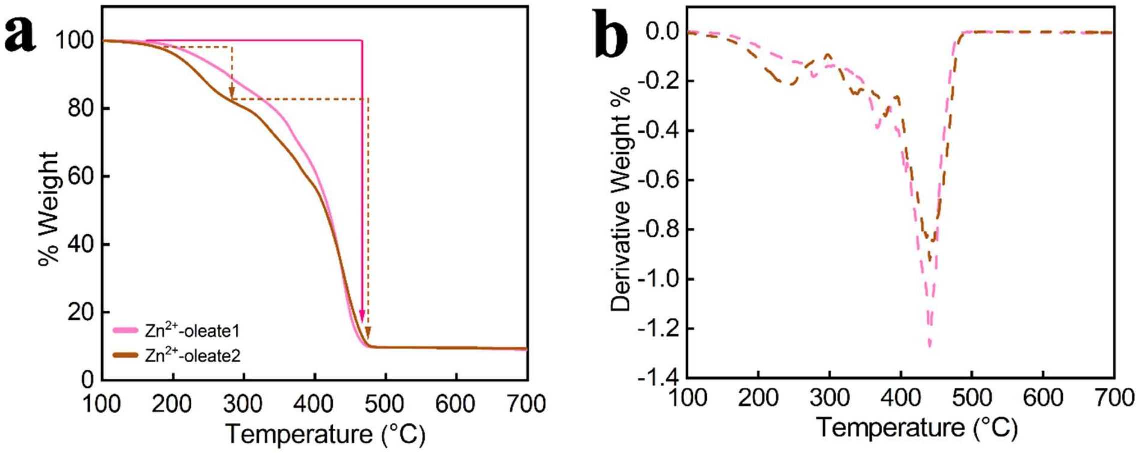

Figure S4. (a) TGA and (b) DTG curves of zinc oleate precursors as indicated.

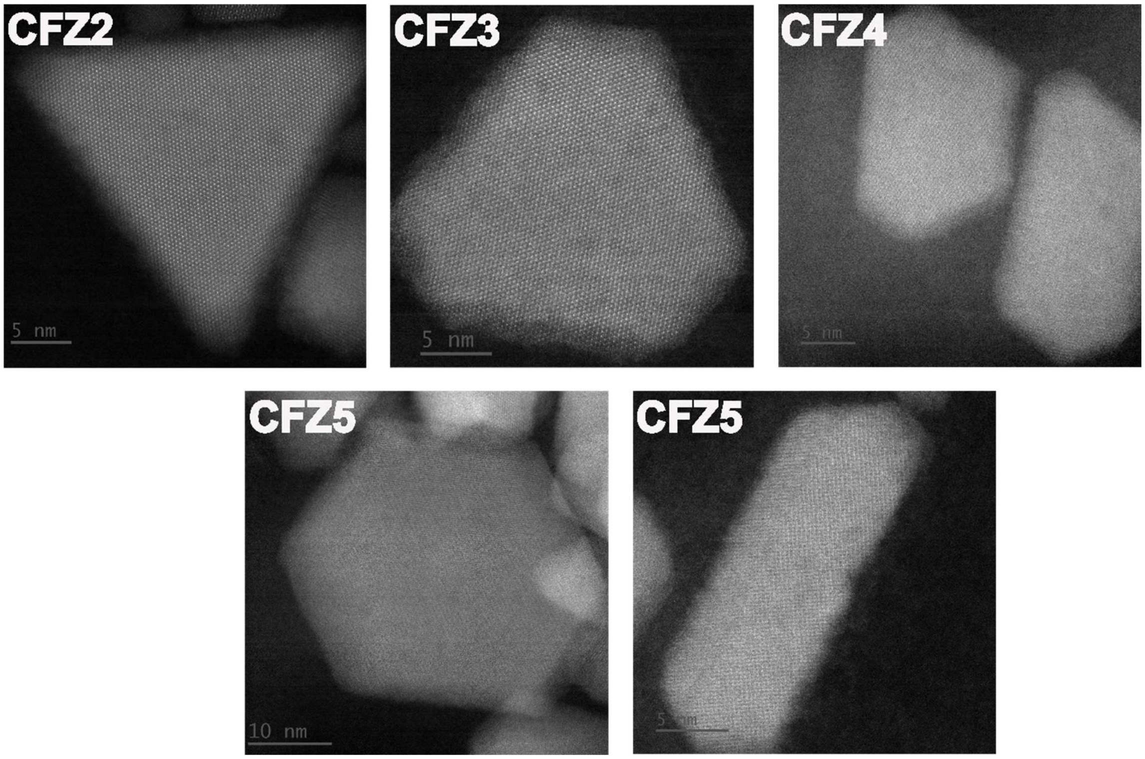

Figure S5. HAADF-STEM images of kinetic morphologies obtained as a result of varrying NaOL to OA ratios.
